# What happens to *Bifidobacterium adolescentis* and *Bifidobacterium longum* ssp. *longum* in an experimental environment with eukaryotic cells?

**DOI:** 10.1186/s12866-023-03179-z

**Published:** 2024-02-19

**Authors:** Dominika Jakubczyk, Katarzyna Leszczyńska, Katarzyna Pacyga-Prus, Dominika Kozakiewicz, Wioletta Kazana-Płuszka, Dominika Gełej, Paweł Migdał, Roksana Kruszakin, Agnieszka Zabłocka, Sabina Górska

**Affiliations:** 1 Laboratory of Microbiome Immunobiology, Hirszfeld Institute of Immunology and Experimental Therapy, Polish Academy of Sciences, Wrocław, Poland; 2 Inter-Departmental Laboratory of Instrumental Analysis and Preparation, Hirszfeld Institute of Immunology and Experimental Therapy, Polish Academy of Sciences, Wrocław, Poland

**Keywords:** *Bifidobacterium*, Probiotic, Redox activity, Membrane potential, Viability, Enzymatic activity

## Abstract

**Background:**

The impact of probiotic strains on host health is widely known. The available studies on the interaction between bacteria and the host are focused on the changes induced by bacteria in the host mainly. The studies determining the changes that occurred in the bacteria cells are in the minority. Within this paper, we determined what happens to the selected *Bifidobacterium adolescentis* and *Bifidobacterium longum* ssp. *longum* in an experimental environment with the intestinal epithelial layer. For this purpose, we tested the bacteria cells' viability, redox activity, membrane potential and enzymatic activity in different environments, including CaCo-2/HT-29 co-culture, cell culture medium, presence of inflammatory inductor (TNF-α) and oxygen.

**Results:**

We indicated that the external milieu impacts the viability and vitality of bacteria. *Bifidobacterium adolescentis* decrease the size of the live population in the cell culture medium with and without TNF-α (*p* < 0.001 and *p* < 0.01 respectively). In contrast, *Bifidobacterium longum* ssp. *longum* significantly increased survivability in contact with the eukaryotic cells and cell culture medium (*p* < 0.001). *Bifidobacterium adolescentis* showed significant changes in membrane potential, which was decreased in the presence of eukaryotic cells (*p* < 0.01), eukaryotic cells in an inflammatory state (*p* < 0.01), cell culture medium (*p* < 0.01) and cell culture medium with TNF-α (*p* < 0.05). In contrast, *Bifidobacterium longum* ssp. *longum* did not modulate membrane potential. Instead, bacteria significantly decreased the redox activity in response to milieus such as eukaryotic cells presence, inflamed eukaryotic cells as well as the culture medium (*p* < 0.001). The redox activity was significantly different in the cells culture medium vs the presence of eukaryotic cells (*p* < 0.001). The ability to β-galactosidase production was different for selected strains: *Bifidobacterium longum* ssp. *longum* indicated 91.5% of positive cells, whereas *Bifidobacterium adolescentis* 4.34% only. Both strains significantly reduced the enzyme production in contact with the eukaryotic milieu but not in the cell culture media.

**Conclusion:**

The environmental-induced changes may shape the probiotic properties of bacterial strains. It seems that the knowledge of the sensitivity of bacteria to the external environment may help to select the most promising probiotic strains, reduce research costs, and contribute to greater reproducibility of the obtained probiotic effects.

**Supplementary Information:**

The online version contains supplementary material available at 10.1186/s12866-023-03179-z.

## Highlights

• External/experimental milieu impacts the viability and vitality of bacteria.

• Bacteria differ in their response to the environments.

• Environmental-induced changes may shape the probiotic properties of bacterial strains.

• The knowledge of the sensitivity of bacteria to the external environment may help to select the most promising probiotic strains, reduce research costs, and contribute to greater reproducibility of the obtained probiotic effects.

**Supplementary Information:**

The online version contains supplementary material available at 10.1186/s12866-023-03179-z.

## Background

The widely accepted definition says that probiotic bacteria are live microorganisms that, when supplemented in an appropriate dose, have a beneficial effect on the host. Many studies show that selected bacterial strains possess the ability to modulate the course of the disease and alleviate its symptoms. *Bifidobacterium* seems to be particularly relevant for human health. They are dominant in healthy, breastfed infants but their percentage changes in adulthood and decreases in old age. Many bifidobacterial strains own probiotic features. They modulate gut barrier homeostasis and shape the immune system. They produce vitamins as well as metabolites such as short-chain fatty acids protecting against pathogenic bacteria [1, 2]. However, available studies indicate that the beneficial effect is a variable trait. The effectiveness of probiotics is linked to the length of use. Longer supplementation is associated with strengthening the intestinal barrier, elongated periods of remission among patients with chronic inflammatory diseases such as inflammatory bowel disease, improvement in clinical scores, and many others [3–5]. The effectiveness of supplemented probiotics is less pronounced when bacteria are administered during the acute inflammatory state [6]. It has also been proven that the genetic background of the host affects the probiotic’s properties of bacteria [7]. Moreover, the same bacterial strains, available commercially, can induce different effects: beneficial, harmful, or neutral [8, 9]. The existing data reporting host-bacterial interactions mainly focus on the assessment of their effect on eukaryotic cells. In turn, reports assessing changes in bacterial cells are in the minority, but, those that exist, clearly show that bacteria are malleable organisms that actively recognize the changeable environment [10]. How these internal bacterial alterations shape their properties, especially in a probiotic context, is unclear. Some external stimuli may affect the function of the bacteria cells, foreclosing them from functioning desirably.

Factors influencing the retention of the bacteria may come from the host but not solely. The research on probiotic strains is based on many experiments leading to the commercialization of the supplement formula. In the research aspect, the in vitro phase allows for assessing the bacterial effects on cell lines. Obtaining promising results allows for further research steps. The following stages include animal models and then clinical trials. Each of the phases provides a different stimulus, for example, bacterial preparation procedures, oxygen availability, cell line culture medium, digestive juices from living organisms, other bacterial species present in the intestine, the host's genetic background, their diets, etc. All of these factors can significantly affect the vitality of tested bacterial strains. Therefore, knowledge about the changes that occur in bacterial cells and how these alterations impact the viability and vitality of selected strains seems to be essential in the entire research process. Consequently, we hypothesized that the environment shapes the bacteria's properties which in turn can modulate the effect exerted. To assess this statement, we selected two strains, which are studied in our laboratory as potentially probiotic ones. Since they differ in general appearance at standard bacterial grown (one of the strains produces a mucus-like layer, while the other strain does not have such a feature), we decided to determine their individual properties by placing the bacteria in different experimental environments and conducting a series of examinations. Based on the redox activity, membrane potential, survival assessment and finally enzymatic activity, we defined the differences that occurred in bacteria’s cells in response to contact with the experimental milieus (including eukaryotic cells, cell line medium, and oxygen presence).

## Results

### The cell proliferation analysis indicates that selected bacteria are not harmful to the intestinal epithelial barrier

**Fig. 1:**
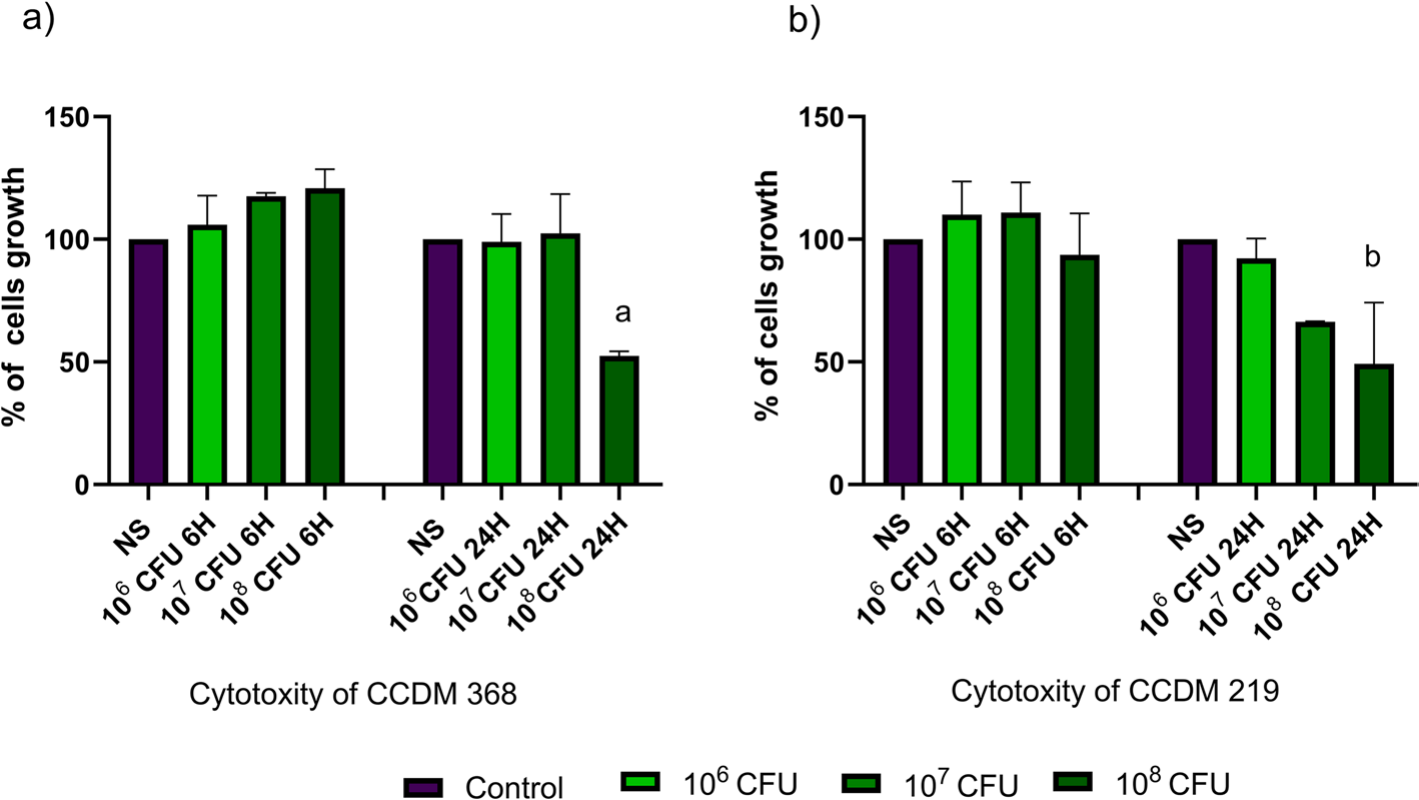
The cell proliferation analysis of CaCo2/HT29 cells after contact with the selected bacterial strains for. **a** CCDM 368 impact after 6 h and 24 h CaCo2/HT29 stimulation; **b** CCDM 219 impact after 6 h and 24 h CaCo2/HT29 stimulation. The statistical analysis was based on the One-way ANOVA test. Significance vs non-stimulated samples: a*, b*. Non-stimulated samples (NS) were marked in purple and stimulated by bacteria in green

First, we determined whether the selected *Bifidobacterium* strains are harmful to the co-culture of CaCo2/HT29 cells. For this purpose, we used the SRB test and checked the impact of bacteria after 6 h and 24 h of co-stimulation (Fig. 1). We proceeded with the amount of bacteria 10^6^, 10^7^, and 10^8^ CFU which ensured a ratio of bacteria cells to the number of eukaryotic cells 1: 33, 1: 333 and 1: 3333 respectively. After 6 h of stimulation, neither of strains CCDM 368 and CCDM 219 impacted the growth of the CaCo2/HT29 cells, nevertheless, after 24 h of stimulation, both strains at the amount of 10^8^ CFU, significantly decreased the number of eukaryotic cells (*p* < 0.05). Additionally, the tendency to reduce eukaryotic cell growth was observed. Therefore, in further experiments, we decided to use the number of bacterial cells and eukaryotic cells ensuring a ratio of 1:333.

### TEM imaging revealed no differences in bacterial cell morphology after contact with the eukaryotic cell

**Fig. 2:**
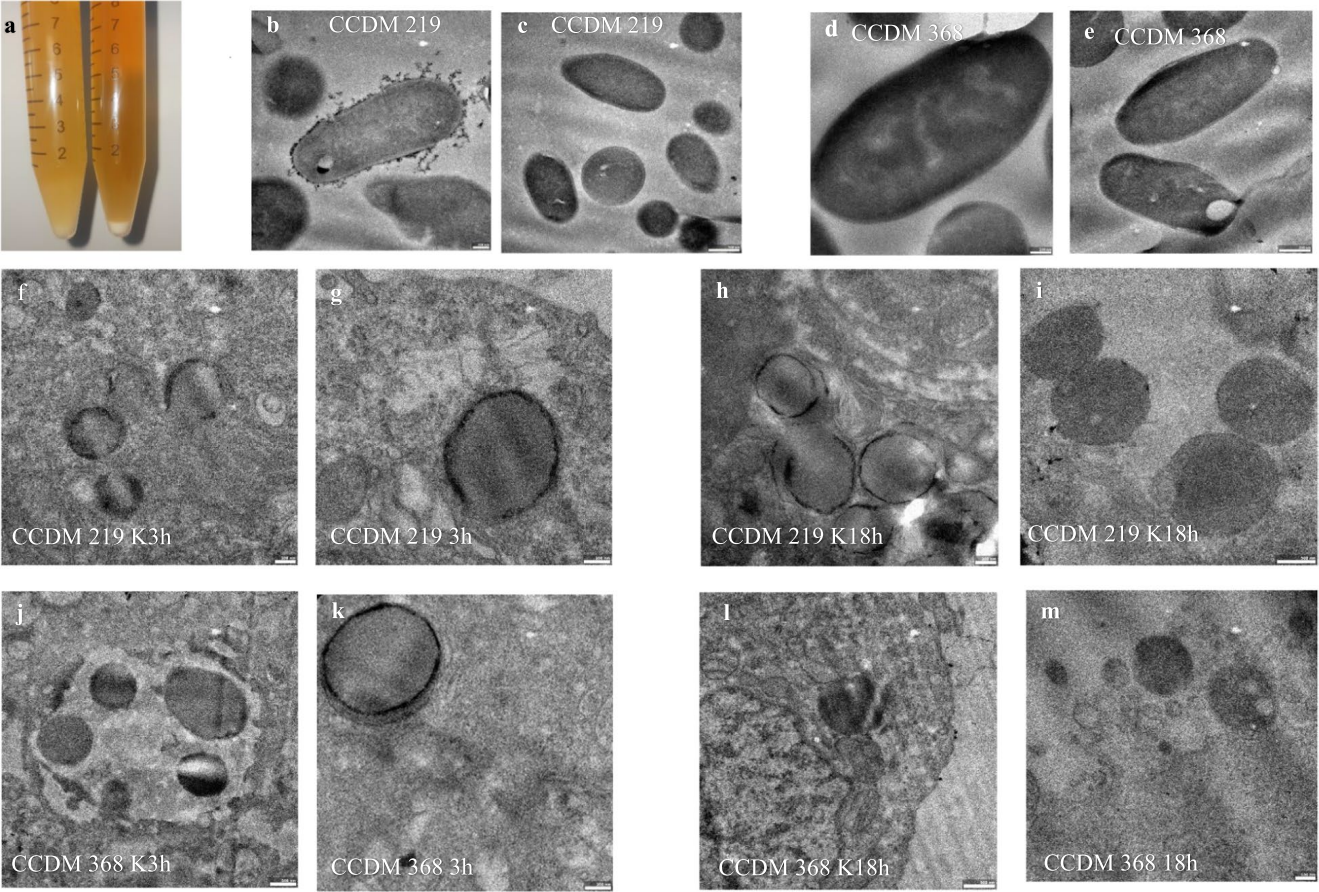
Morphology of strains CCDM 368 and CCDM 219; **a** macroscopic appearance of bacterial direct from MRS overnight culture in anaerobic condition, on the right strain CCDM 368, on the left strain CCDM 219; TEM imaging of strain: **b**-**c** CCDM 219, **d**-**e** CCDM 368 directly from MRS overnight culture in anaerobic condition (baseline); TEM imaging of strain: **f**-**i** CCDM 219, **j**-**m** CCDM 368 in different environments (in co-culture with CaCo2/HT29 cells); K3h: co-culture CaCo2/HT29 with selected bacteria, without inflammatory state (control), 3 h: CaCo2/HT29 with inflammatory state induced 3 h after bacteria addition, K18h: co-culture CaCo2/HT29 with selected bacteria, without inflammatory state (control), 18 h: CaCo2/HT29 with inflammatory state induced 18 h before bacteria addition

Based on the above findings, we decided to proceed with further experiments with the use of bacteria and co-culture of human epithelial cells of intestinal origin. The first question asked was 'What happens to the bacterial cells in an experimental environment with eukaryotic cells?' We started with a series of TEM imaging. The selected bacterial strains were imaged after overnight culture in MRS broth (anaerobic conditions; baseline) and then, in the co-culture with eukaryotic cells in different environments. As is shown in Fig. 2a) bacteria differ in macroscopic appearance in MRS broth at the basic state. CCDM 368 strain (on the right) created a cohesive pellet, whereas the distinguishing feature of CCDM 219 (on the left) was a fuzzy growth. The TEM imaging of the baseline samples (Fig. 2b-e) indicated that both bacteria have oval or round shapes. CCDM 219 strain indicated an additional layer around the cells, however, it was reduced after contact with eukaryotic cells. The shape differences between both strains were not observed (Fig. 2f-m). Therefore, we decided to check whether dwelling in these environments was associated with changes in the composition of the basic elements.

### EDS analysis indicates the environmental responsiveness of selected bacteria

**Fig. 3:**
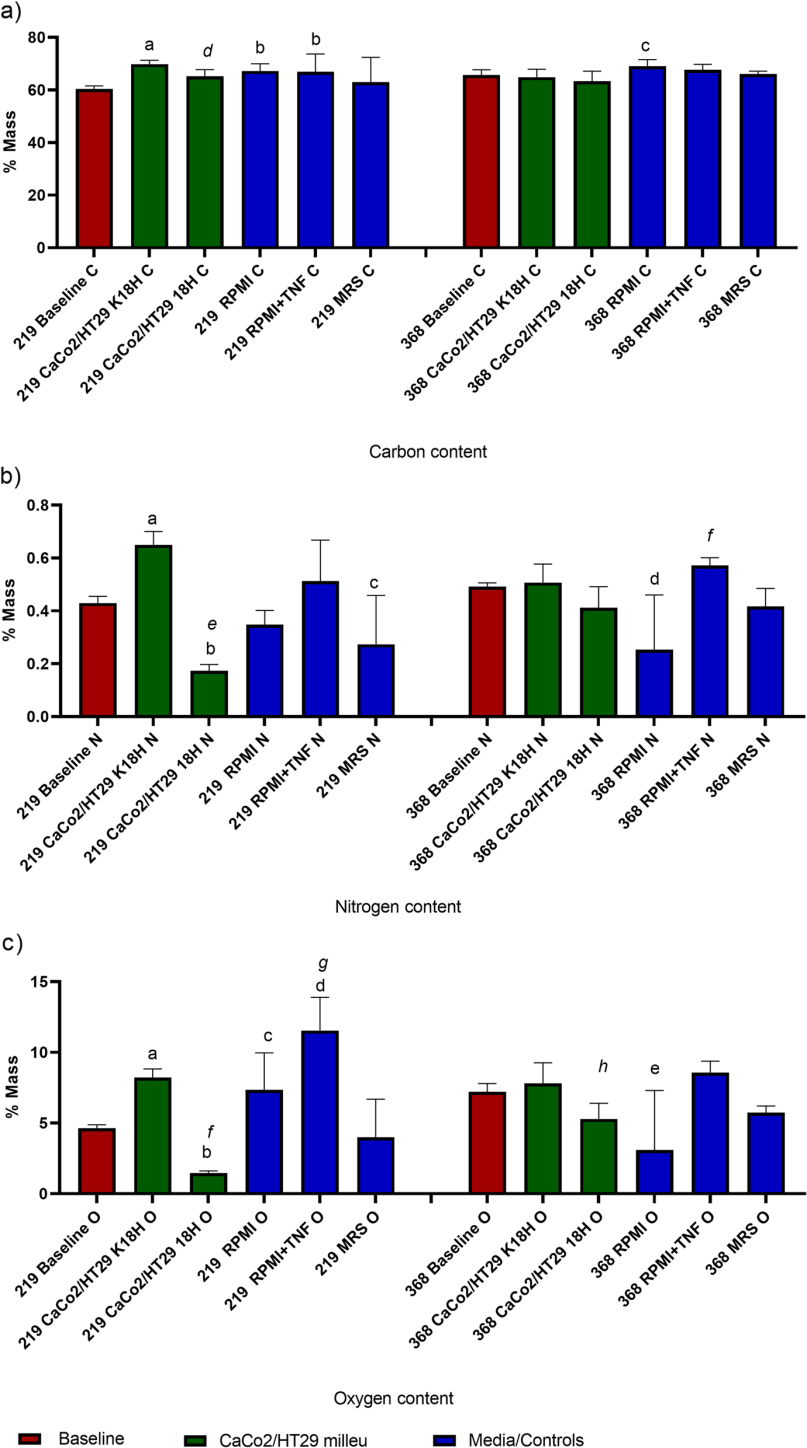
EDS analysis for different elements. The baseline samples were presented in claret colour, the eukaryotic environment was presented in green, and the control samples in blue. The analysis of baseline vs samples was based on the One-way ANOVA. The differences between groups K18h vs 18 h and RPMI vs RPMI + TNF-α were analysed by paired t-test. **a** Carbon contents in bacteria placed in various environments. Significance for strain CCDM 219 versus baseline: a **, b*; K18h vs 18 h: *d* ***. Significance for strain CCDM 368 versus baseline: c*. **b** Nitrogen contents in bacteria placed in various environments. Significance for strain CCDM 219 versus baseline: a ***, b ****, c*; K18h vs 18 h: e ****. Significance for strain CCDM 368 versus baseline: d ****, RPMI vs RPMI + TNF-α: f *. **c** Oxygen contents in bacteria placed in various environments. Significance for strain CCDM 219 versus baseline: a ***, b **, c*, d****; K18h vs 18 h: f ****; RPMI vs RPMI + TNF-α: g*. Significance for strain CCDM 368 versus baseline: e ***, K18h vs 18 h: h *

Since the 3 h contact with the inflammatory environment (experimental model 2) could not fully reflect the occurred cell changes, therefore we decided to base the experiment on model 1 (Active inflammatory state) exclusively. Analysis of the elements’ composition indicated changes in both strains depending on the environment. In comparison with the baseline state, bacteria differed in carbon (C), oxygen (O) and nitrogen (N) contents. In comparison with the baseline samples, strain CCDM 219 indicated a significant increase in carbon presence in samples K18h (*p* < 0.01), RPMI (*p* < 0.05) and RPMI + TNF-α (*p* < 0.05). A significant difference was noted also for samples K18h vs 18 h (*p* < 0.01). The results obtained for nitrogen indicated differences for baseline vs K18h (*p* < 0.001), vs 18 h (*p* < 0.001) and vs MRS (*p* < 0.05). A significant difference was noted for samples K18h vs 18 h (*p* < 0.001). The oxygen presence differed for baseline vs K18h (*p* < 0.001), vs 18 h (*p* < 0.01), vs RPMI (*p* < 0.05) and vs RPMI + TNF-α (*p* < 0.001). A significant difference was indicated for samples K18h vs 18 h (*p* < 0.0001) and RPMI vs RPMI + TNF-α (*p* < 0.05). Strain CCDM 368 indicated different elements distribution in individual samples. In comparison with the baseline samples, strain CCDM 368 indicated a significant increase in carbon contents only in RPMI samples (*p* < 0.05). The changes in nitrogen were noted for baseline vs RPMI (*p* < 0.001). A significant difference was noted for samples RPMI vs RPMI + TNF-α (*p* < 0.05). Whereas the oxygen presence differed for baseline vs RPMI (*p* < 0.05) and K18h vs 18 h (*p* < 0.05). The detailed data is shown in Fig. 3.

### Bacterial live-dead analysis indicated that the available milieu impacts the CCDM 219 and CCDM 368 strains' survival

**Fig. 4:**
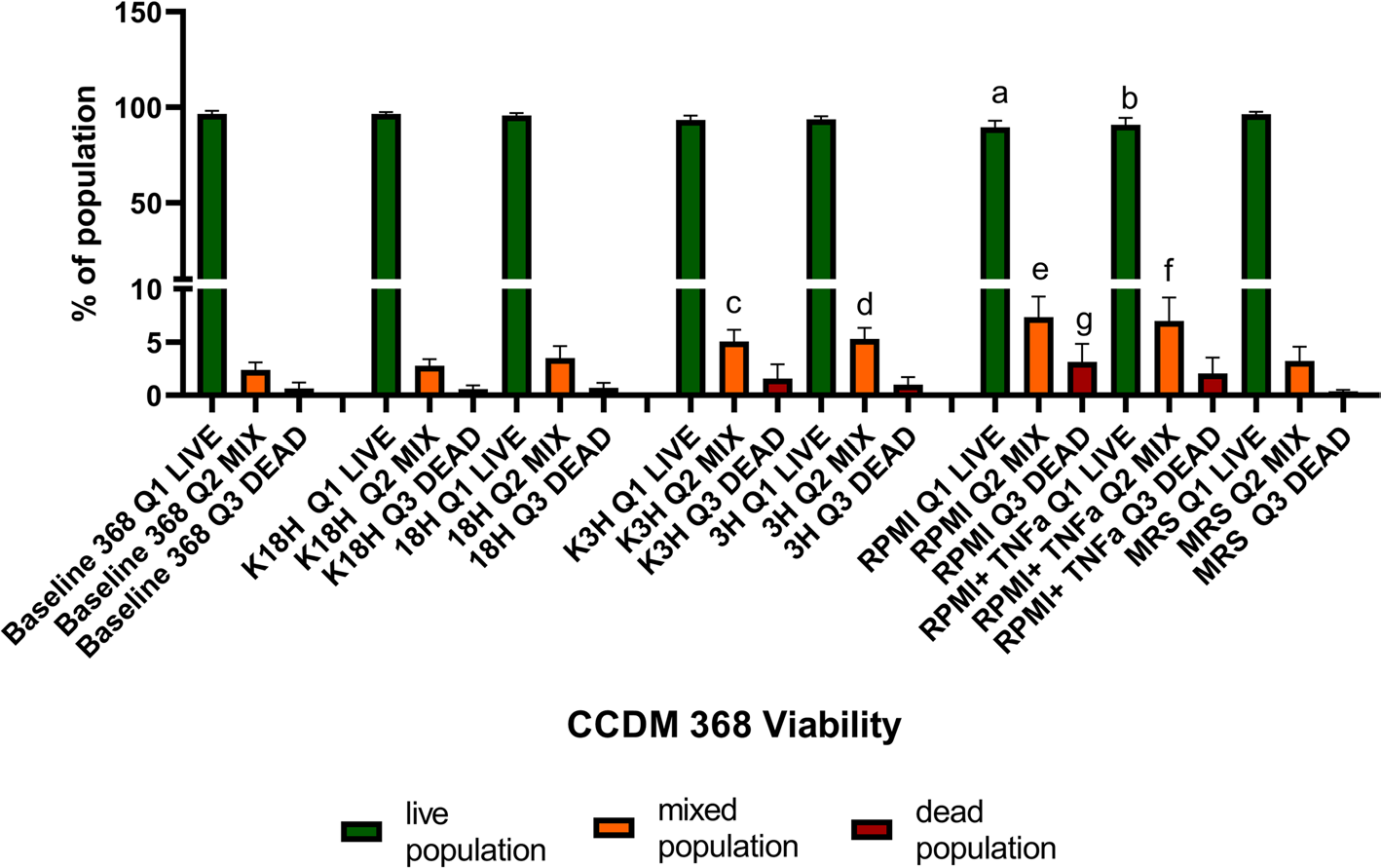
The survival (viability analysis) of *Bifidobacterium* CCDM 368 strain in different environments. The live population was presented in green, the mixed one in orange, and the dead one in red. The analysis of baseline vs samples was based on the One-way ANOVA. Significance: in live population: a ***, b**; in mixed population: c*, d**, e**** f ***, in dead population: g **

**Fig. 5:**
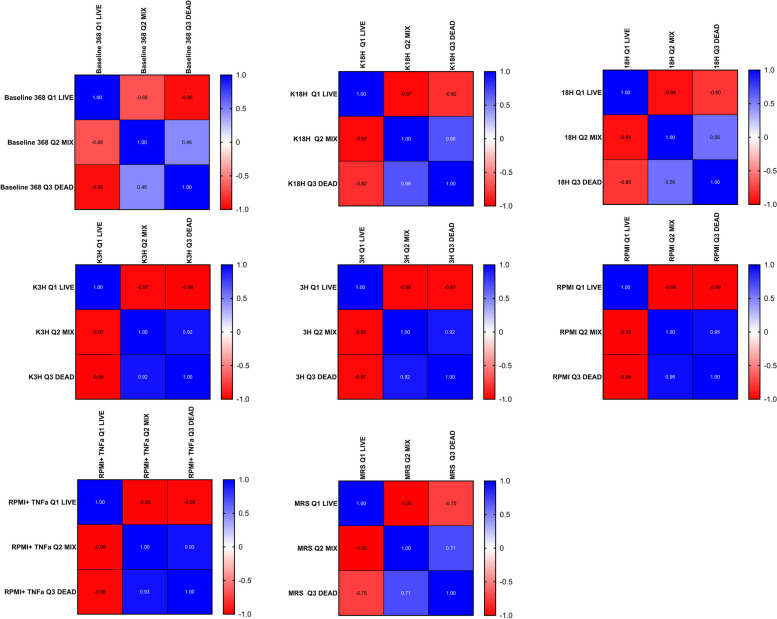
Pearson's linear correlation analysis for different groups (baseline, an active inflammatory state model, protection against inflammatory state, and appropriate controls). Interpretation of the correlation coefficient r strength: lack or very weak correlation *r* = 0–0.3; moderate: 0.3–0.5, strong: 0.5–0.7; very strong: 0.7–1.00)

*Bifidobacterium* is an anaerobic bacteria, which, to maintain its viability, demands strictly defined culture conditions. Traditionally these bacteria are cultivated in MRS broth supplemented with L-cysteine and in the absence of oxygen. On the other hand, the eukaryotic cultures are maintained in the presence of oxygen and 5% CO_2_, as well as a cell’s specific media, enriched in FBS and even antibiotics. The ‘bacteria- eukaryotic cells’ based experiments usually rely on the placement of the bacteria in a foreign, eukaryotic cell culture environment. If the beneficial effect evoked by *Bifidobacterium* is mediated by its metabolic activity, retention of the live population seems to be crucial. Therefore, the asked question was: How do these unfavourable surroundings impact the *Bifidobacterium* strains' survivability? To answer it, we washed the bacteria from the MRS broth and suspended them in PBS first. Then, we added 10^8^ CFU/ml to the wells with CaCo2/HT29 co-culture according to assumptive models (‘Active inflammatory state’ and ‘Protection against inflammatory state’). After 6 h stimulation, we determined the presence of live and dead populations based on the flow cytometric measurement. As shown in Figs. 4 and 7, selected bacterial strains were characterised by different features. Strain CCDM 368 (Fig. 4, Additional file 1) seems to be stable in culture, and the baseline (bacteria culture in MRS + L-cysteine broth in anaerobic conditions) indicated a predominance of the live population (mean value 96.62%). A significant decrease in the live population was noted for bacteria in RPMI (up to 89.4%) and RPMI with the addition of TNF-α (up to 90.9%) (*p* < 0.001 and *p* < 0.01 respectively). The cellular environment did not impact the size of the live population. The dead population increased in RPMI alone environment (baseline mean value was 0.65%, whereas RPMI mean value was 3.14%, *p* < 0.01), and some tendency to its increase was noted also for RPMI with the addition of TNF-α, however without the statistical significance. Interestingly, the third population, called ‘mixed’, was observed in the experiments. The live-dead distinction relies on the different properties of staining factors. Syto 9 interacts with the bacterial nucleic acids, however, it does not bind permanently. PI displaces the Syto 9 staining and creates an undetachable binding. Syto 9 indicates a live population, PI a dead one. The third population, a mixed one, absorbs two dyes. The mixed population was visible in the baseline samples (2.39%), significantly increased in ‘Protection against inflammatory state’ vs baseline (for K3h mean value was 5.06%, for 3 h mean value was 5.2%, *p* < 0.05 and *p* < 0.01 respectively) and in RPMI environments (7.34% for RPMI alone and 6.99% for RPMI with TNF-α, *p* < 0.001 and *p* < 0.001 respectively). There were no differences between K18h vs 18 h, K3h vs 3 h, RPMI vs RPMI with TNF-α and MRS baseline vs MRS in oxygen presence. The analysis of correlation indicated the population-size dependency (strong or very strong for all samples except Baseline Dead vs Baseline Mix population where the correlation was moderate (*r* = 0.46). The detailed data is shown in Fig. 5.

**Fig. 6:**
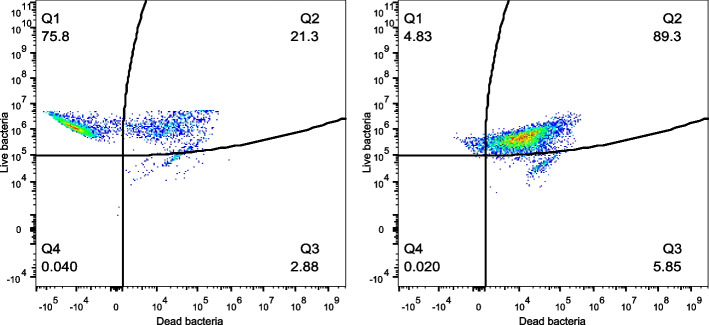
The dots plots examples of the CCDM 219 different states at baseline

**Fig. 7:**
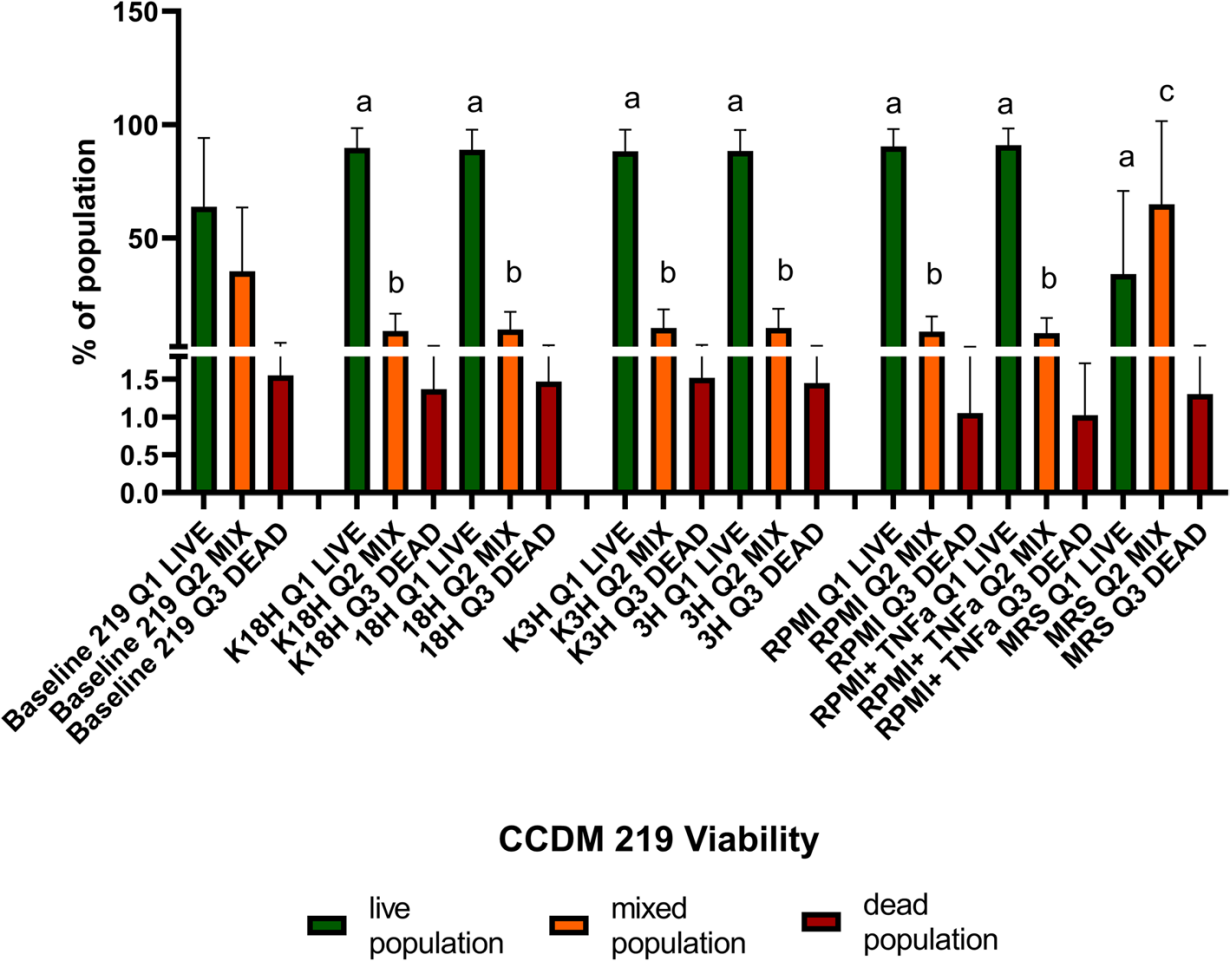
The survival (viability analysis) of *Bifidobacterium* CCDM 219 strain in different environments The live population was presented in green, the mixed one in orange, and the dead one in red. The analysis of baseline vs samples was based on the One-way ANOVA test. Significance: in live population: a *, in mixed population: b*, c**

**Fig. 8:**
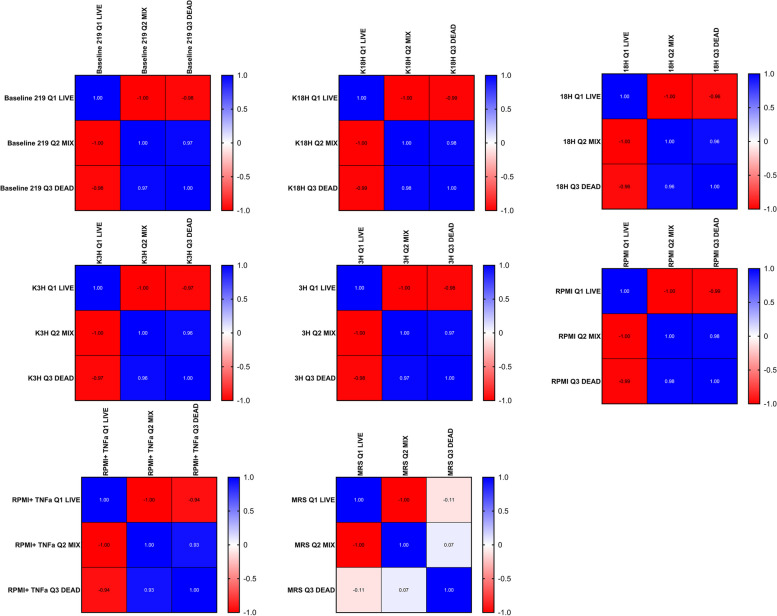
Pearson's linear correlation analysis for different groups (baseline, an active inflammatory state model, protection against inflammatory state, and appropriate controls). Interpretation of the correlation coefficient r strength: lack or very weak correlation *r* = 0–0.3; moderate: 0.3–0.5, strong: 0.5–0.7; very strong: 0.7–1.00)

In comparison to the CCDM 368 strain, the CCDM 219 was characterised by different properties. The baseline samples indicated the fluctuation between the passages (Fig. 6). The obtained mean value for the live population in baseline was 63.78%, mixed population: 35.35%, and dead population 1.55%. However, the proportion varied between experiments. The significant differences between the baseline and experimental samples for the live and mixed populations were noted. For all groups, a significant increase in live population was observed (baseline vs K18h: *p* < 0.05; vs 18 h: *p* < 0.05; vs K3h: *p* < 0.05; vs 3 h: *p* < 0.05; vs RPMI: *p* < 0.05; vs RPMI + TNF-α: *p* < 0.05; and vs MRS: *p* < 0.05). Simultaneously, a significant decrease in mixed population in all groups was noted (baseline vs K18h: *p* < 0.05; vs 18 h: *p* < 0.05; vs K3h: *p* < 0.05; vs 3 h: *p* < 0.05; vs RPMI: *p* < 0.05; vs RPMI + TNF-α: *p* < 0.05; and, except the MRS control group, where the mixed population increased significantly (baseline vs MRS: *p* < 0.01). No significance was observed in a dead population. There were no differences between K18h vs 18 h, K3h vs 3 h, and RPMI vs RPMI with TNF-α. The detailed data is shown in Fig. 7 and Additional file 1. Pearson's linear correlation analysis indicated a strong dependency between the populations in the baseline as well as in the groups K18h, 18 h, K3h, 3 h, RPMI, and RPMI + TNF-α. The increase in live population negatively correlated with the decrease in mixed or dead population (the bigger the live population, the smaller dead or mixed populations). Moreover, the mixed population positively correlated with the dead population (the bigger the mixed population, the bigger the dead population in the samples). However, this pattern was not confirmed in the MRS control samples (oxygen presence). In this case, the live population strongly correlated with the mixed population, but no dependency was noted for the dead group (Fig. 8).

### Strain CCDM 219 indicates significant changes in redox activity in response to the foreign environments

**Fig. 9:**
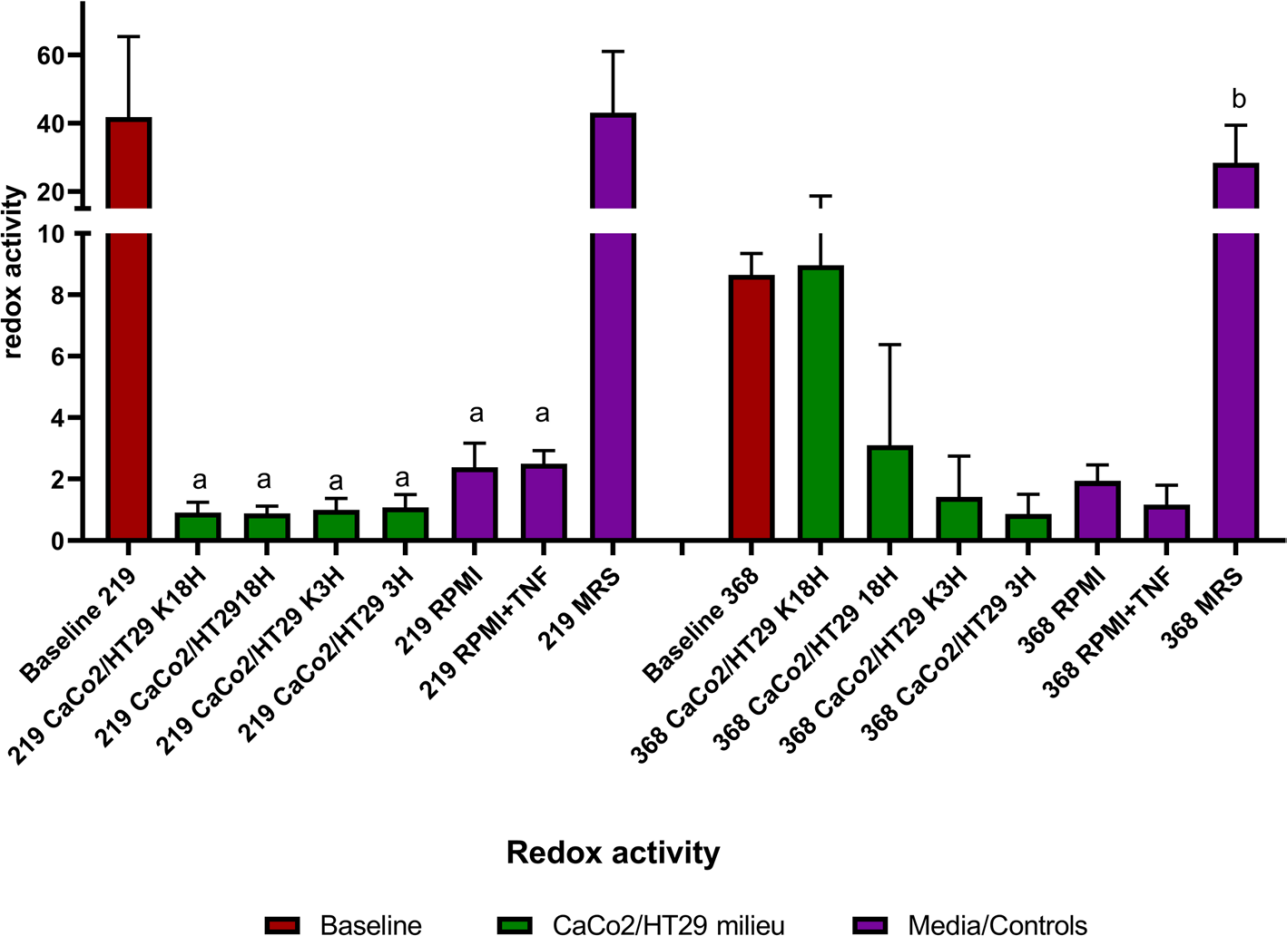
The CCDM 219 and CCDM 368 strains’ redox activity in different environments. The baseline samples were presented in claret, the eukaryotic milieu in green, and the control samples in purple. The analysis of baseline vs samples was based on the One-way ANOVA test. Significance: CCDM 219 baseline vs remaining groups (a****), CCDM 368 baseline vs remaining groups ( b****)

We indicated that the selected bacteria can survive in a foreign milieu, however, the size of live, mixed and dead populations underwent strain- and environment-dependent changes. Our attention was attracted by the oxygen bacterial response. In an aspect of bacterial-based supplement production, some tolerogenic properties of probiotic bacteria are desired. As shown in Figs. 4 and 7, the selected bacteria are characterised by different oxygen resistance. The presence of oxygen did not shape the viability of strain CCDM 368 (MRS control sample vs baseline). On the contrary, strain CCDM 219 indicated a significant decrease in the live population and an increase in the dead one when suspended in MRS broth in oxygen presence. Therefore, we decided to check bacterial vitality, understood as a ‘state of being strong and active’. For this purpose, we determined bacterial respiratory activity. The commercially available redox sensor test evaluates the respiratory activity based on the bacterial properties to the reduction of CTC. The ‘healthy’ bacteria can reduce CTC in an insoluble red fluorescence product. This ability is impaired or stopped among ‘unhealthy’ or dead bacteria. As shown in Fig. 9, the selected bacterial strains differ in their properties. The strain CCDM 219 at a baseline and MRS control indicated a high redox potential. However, the redox activity significantly decreased in bacteria placed in foreign environments (baseline vs K18h, 18 h, K3h, 3 h, RPMI, and RPMI + TNF-α: *p* < 0.001). The RPMI and RPMI with TNF-α indicated a significant increase in redox activity in comparison with the cellular components (RPMI vs K18h: *p* < 0.001; vs 18 h: *p* < 0.001; vs K3h: *p* < 0.001; vs 3 h: *p* < 0.001; RPMI with TNF-α vs K18h, 18 h, K3h, 3 h: *p* < 0.001). There were no differences between K18h vs 18 h, K3h vs 3 h, and RPMI vs RPMI with TNF-α.

Strikingly, strain CCDM 368 indicated different properties. The baseline samples indicated some redox activity, however significantly lower in comparison with CCDM 219 (*p* < 0.001). The analysis of CCDM 368 baseline vs MRS control samples indicated a significant increase in redox activity in the oxygen milieu (*p* < 0.001). The redox activity was comparable between the baseline sample and K18h and decreased in the remaining groups, however without any significance. There were no differences between K18h vs 18 h, K3h vs 3 h, and RPMI vs RPMI with TNF-α but the tendency to decrease the redox activity in an inflamed environment was visible. The detailed data is shown in Fig. 9 and Additional file 2.

### Strain CCDM 368 but not CCDM 219 indicates significant changes in membrane potential in response to the foreign environments

**Fig. 10:**
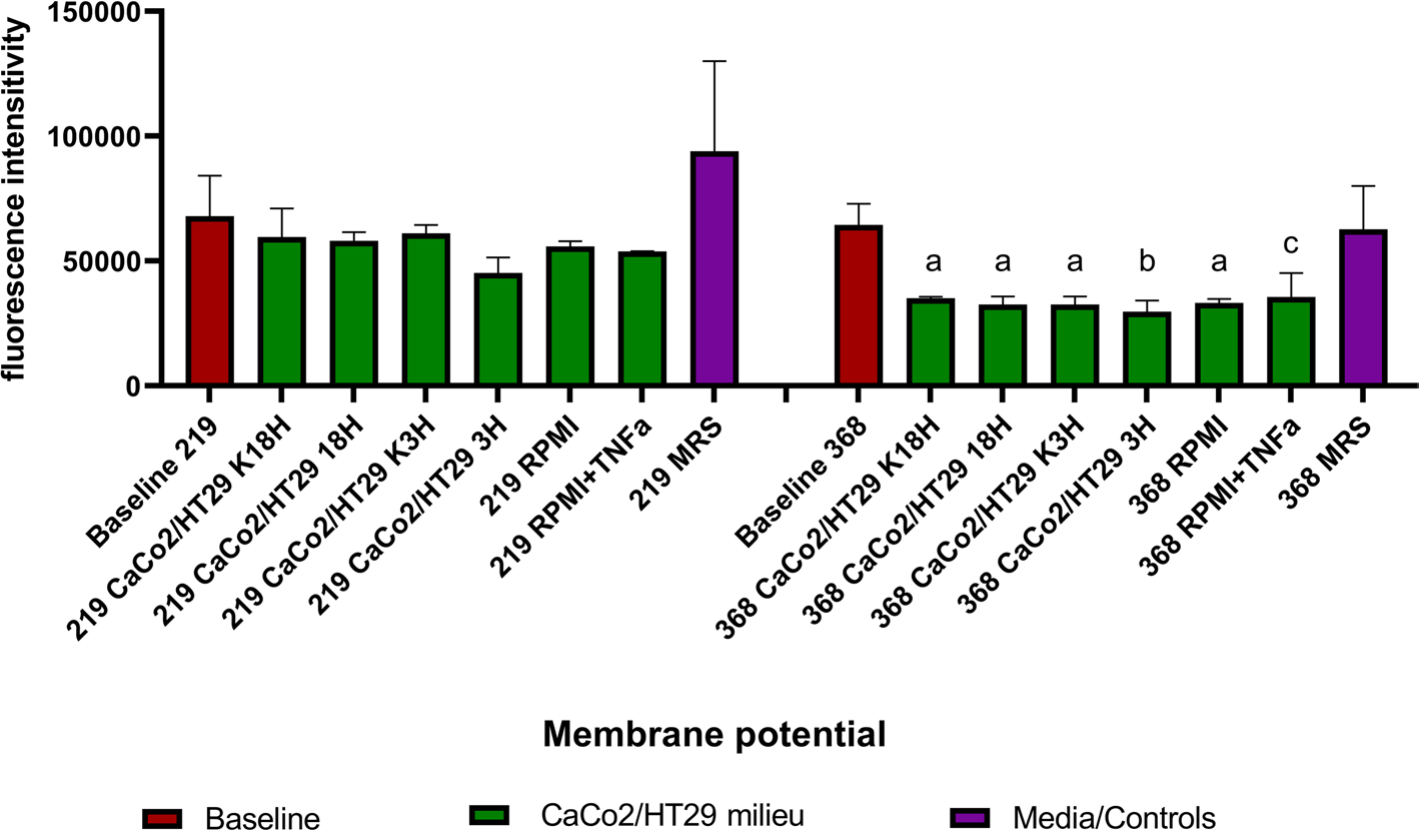
The CCDM 219 and CCDM 368 strains membrane potential maintenance. The baseline samples were presented in claret, the eukaryotic milieu in green, and the control samples in purple. The analysis of baseline vs samples was based on the One-way ANOVA test. Significance: a**, b***, c*

Concerning bacterial vitality, we decided to determine the changes in bacterial membrane potential as well. The commercially available test is based on the carbocyanine dye DiOC_2_(3) which indicates a red fluorescence linked with a dye self-association caused by larger membrane potential. The attenuated membrane potential is linked with the lower vitality of bacteria. Since the DiOC_2_(3) dye is characterised by crystallisation properties, to avoid cytometer damage, we decided to check the carbocyanine self-association with the use of the microplate fluorescence reader. As shown in Fig. 10, the selected strain differs in the membrane potential maintenance. The strain CCDM 219 did not indicate a significant alteration of the membrane potential in different environments. There were no differences between K18h vs 18 h, K3h vs 3 h, and RPMI vs RPMI with TNF-α. However, strain CCDM 368 reacted in a completely different manner. The eukaryotic milieu, as well as the cell culture medium, led to a decrease in membrane potential (baseline vs K18h *p* < 0.01, vs 18 h *p* < 0.01, vs K3h *p* < 0.01, vs 3 h *p* < 0.001, vs RPMI *p* < 0.01, and RPMI + TNF-α *p* < 0.05). Bacteria placed in MRS broth in oxygen presence (control) did not change the membrane potential in comparison with the baseline. There were no differences between K18h vs 18 h, K3h vs 3 h, and RPMI vs RPMI with TNF-α. The detailed data of membrane potential as well as bacterial response to the environment with and without antibiotics is shown in Fig. 10 and Additional file 3 respectively.

### Strain CCDM 368 and CCDM 219 differ in β-galactosidase (β-gal) production and the cellular environment shapes the bacterial enzymatic activity

**Fig. 11:**
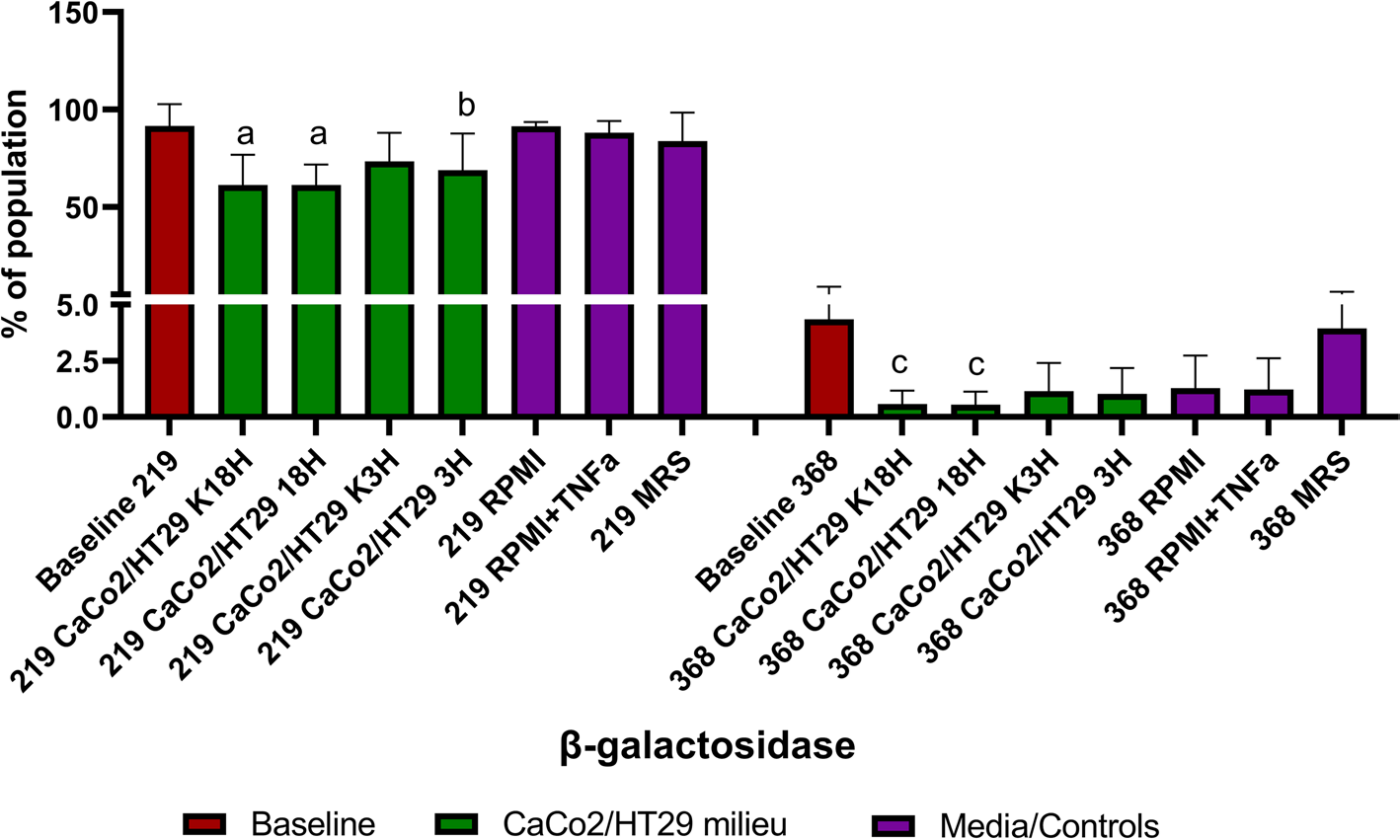
Percentage of cells which were able to produce β-gal in different environments. The baseline samples were presented in claret, the eukaryotic milieu in green, and the control samples in purple. The analysis of baseline vs samples was based on the One-way ANOVA test. Significance: for CCDM 219: a***, b*, for CCDM 368: c*

Finally, we decided to check the ability of bacteria to produce the enzyme, β-galactosidase, when exposed to different environments. As is shown in Fig. 11, the bacteria differed in their production. Up to 91.5% of bacteria strain CCDM 219 produced the enzyme at baseline state. This value was reduced in the eukaryotic milieu (baseline vs K18h: mean value 61.15%, *p* < 0,001; vs 18 h: mean value 61.24%, *p* < 0.01, vs K3h: mean value 73.32, ns; vs 3 h, mean value 68.75, *p* < 0.05). The significant differences for the control samples were not observed and their mean values were comparable with the baseline (baseline vs RPMI: mean value 91.4%, ns; vs RPMI + TNF-α: mean value: 88.03%, ns; vs MRS: mean value 83.7%, ns). There were no differences between K18h vs 18 h, K3h vs 3 h, and RPMI vs RPMI with TNF-α. However, some tendency in the pairs of the group was noted. The highest level of cells produced β-gal was observed for MRS and fresh RPMI medium-based control. The pair of tested groups K18h and 18 h indicated the lowest percentage of cells able to produce β-gal. Strain CCDM 368 also produced β-gal, however, the baseline mean value was lower in comparison with strain 219 (4.34%). A significant decrease in comparison with baseline samples was observed for K18h and 18 h groups (baseline vs K18h: mean value 0.57%, *p* < 0.05; vs 18 h: mean value 0.54%, *p* < 0.05). Some tendency to decrease the number of β-gal positive cells was observed for the K3h (mean value 1.15%), 3 h (mean value 1.03%), RPMI (mean value 1.28%), RPMI with TNF-α (mean value: 1.27%), however, without any significance. The mean value of the MRS control sample (3.94%) was comparable with the baseline value. The detailed data is shown in Fig. 11 and Additional file 4.

## Discussion

In recent years, the term 'therapeutic microbiology' has been gaining popularity [11]. The beneficial effect can be mediated by bacterial components (surface proteins, peptidoglycan, etc.), as well as by their metabolites [12]. Many bacteria are credited with probiotic properties that contribute to the maintenance of the host's health. Dozens of experimental studies indicate that some bacterial strains increase the integrity of the intestinal epithelial barrier, alleviate inflammation, reduce disease symptoms and many others [13–15]. Generally, these positive observations are reflected in clinical trials as well [16, 17]. However, apart from studies proving the effectiveness of selected bacteria, some data is less enthusiastic and indicates that the same strains are poorly functional or even harmful to the host. Biagioli et al. [8] determined the beneficial properties of two batches of the same probiotic product and found diversified results. One of the batches had a beneficial impact on the IBD and attenuated the disease indicators in the murine model of colitis, whereas the other one even worsened the disease course by increasing the epithelial permeability. Similarly, Palumbo et al. [9] indicated opposite properties of commercially available VSL#3 probiotic mixture derived from different manufacturers on the CaCo-2 epithelial barrier model. Kennedy et al. [18] reported that *Lactiplantibacillus plantarum* species 299 failed to improve gut permeability in rat’s model of colitis, whereas some other studies confirmed the beneficial impact on the inflamed human colon [19]. The above issues and discrepancies led us to conclude that the baseline viability and vitality of the bacteria used in the experiment, often neglected, are crucial for capturing the probiotic effect. In fact, the immune system can distinguish between live and dead bacteria and respond differently to those. For instance, the murine macrophages and dendritic cells react more intensely to the live bacteria. The interaction is mediated by TLR (Toll-like receptors) and leads to NLRP3 (NOD-, LRR- and pyrin domain-containing protein 3) inflammasome complex activation, IL-1β releasing, pyroptosis, and increased production of type-I Interferon. The dead bacteria do not activate this path and the host’s response is weaker [20]. Neutrophils in response to the live bacteria activate TLR and formylated peptide receptors 1 and 2 (Fpr1, Fpr2), and produce up to 30-fold higher levels of the Cxcl2 chemokine (C-X-C Motif Chemokine Ligand 2) in comparison to the dead bacteria [21]. Therefore, the recognition of bacteria's live/dead status is essential in conducting experiments, and standardisation of this aspect can make the obtained results more reproducible and reliable. Moreover, apart from examining bacterial initial viability, it also seems important to preserve the alive state during further stimulation. The external milieu can impact the bacterial properties [22, 23] which leads to the modification of their basic features. It was determined that the availability of the carbohydrate sources (glucose, maltose, galactose, sucrose, and lactose) shape the *Lacticaseibacillus rhamnosus* capacity for producing exopolysaccharides (EPS) and depending on the energy sources, the produced EPSs present different molecular mass distributions, chain length, thickness, branching and viscosity [22]. How this environmental-induced modification impacts the host-bacteria interactions is still blurry. Most of the available data is focused on the bacterial impact on the host cell. Our goal was to reverse the model and assess what happens to bacterial cells when they enter a foreign environment including co-culture with eukaryotic cells, complete cell culture medium, inflammatory factors, and oxygen presence. For this purpose, we selected two strains, *Bifidobacterium adolescenti*s CCDM 368 and *Bifidobacterium longum* ssp. *longum* CCDM 219 which difference in macroscopic appearance in MRS traditional culture. In TEM we did not ascertain significant differences in the bacterial appearance, except for the reduction of the mucus-like layer in CCDM 219 strain and the shift to the more round shape. However, this alternation could arise from the preparatory procedures, such as trypsinization of the cells in order to remove them from the culture plate. This procedure can affect the external protein content, and impact the bacterial shape. We also decided to determine the elemental composition of bacteria placed in different milieus. Among others, the carbon, oxygen and nitrogen contents in a bacterial cell reflect its living conditions. Limited access to key elements could be associated with a reduction in bacteria metabolism and growth [24]. Our research showed that both selected *Bifidobacterium* strains actively reacted to environments, which was reflected in their elemental composition. The changes that occurred were differently pronounced by the strains, which indicated the different sensitivity of bacteria to the surrounding milieus. A closer look at the properties of the bacteria revealed further differences. The cytometric analysis of the viability of strains straight from their proper cultivation in the MRS medium (baseline), showed that the bacteria differed in their stability. Strain CCDM 368 was characterised by the consistency and dominance of the live population from passage to passage. In contrast, strain CCDM 219 indicated the fluctuation between the passages and the mixed population was strongly marked. This population could refer to viable but non-culturable (VBNC) bacteria, which remain alive but at a very low metabolic activity level and with inhibited cell division ability. Bacteria enter into this state when they encounter stressful conditions (lack of nutrients, oxygen fluctuation, temperature, etc.), however, removal of these factors may lead to their resuscitation [25–27], which was noticeable in our samples as well. The baseline sample showed the markable mixed population, however, in further experiments bacteria were diluted (to 10^8^ CFU/ml). This could energise dormant cells and increase a live population. Nevertheless, this does not explain the fact that the mixed population was even bigger in the MRS control sample. Oxygen as another stress factor may be overlapping in this case (discussed later). However, on the other hand, the double fluorescence signal can be a result of cell clumps because of the presence of the mucus-like layer in the CCDM 219 strain, biofilm properties of this mucus-layer or even the high membrane potential of this strain [28–30]. The issue of what exactly is a mixed population and what are its properties requires further clarification.

Bacteria differ also in reaction to the contact with CaCo2/HT29 layer and control samples’ milieu. Strain CCDM 368 kept the live population at an unchangeable level in comparison to the baseline, however, increased the mixed population in a model of *Protection against the inflammatory state.* Moreover, a significant decrease in the live population and an increase in the mixed population were noted in control samples with RPMI medium with and without the addition of TNF-α. Bearing in mind that survivability did not change between the groups: K18h vs 18 h, K3h vs 3 h and RPMI vs RPMI + TNF-α, we imply that strain CCDM 368 is sensitive to the complete medium used in the experiment. In model 1 (*An active inflammatory state*), the medium was exchanged 18 h before the administration of bacteria, it had already been 'processed' by eukaryotic cells and therefore it was not harmful to the bacteria cells. In model 2 (*Protection against the inflammatory state*) the medium was changed directly prior to bacterial stimulation, and the eukaryotic cells did not manage to metabolise it. Similarly, in the RPMI controls bacteria were placed directly in a fresh medium. Therefore, the RPMI complete medium impacted the CCDM 368 strain survival after 6 h of contact and this aspect should be considered in cell-line based experiments. However, strain CCDM 219 indicated different properties. The eukaryotic surrounding, as well as the complete RPMI controls medium allowed to increase the percentage of the live population and a simultaneous decrease of the mixed one. Therefore, the cell culture medium seems to be neutral for strain CCDM 219.

Oxygen tolerance is an important aspect of the probiotic-based study. Generally, *Bifidobacterium* strains are considered anaerobic, however, the strains differ in oxygen tolerance. Andriantsoanirina et al. [31] tested the viability of 115 bifidobacterial strains exposed to oxygen within 48 h. The viability was determined as a colony grown on an agar plate (CFU) after oxygen exposure. The authors indicated that, i.e., *B. adolescentis* strains are sensitive (growth of strains was completely inhibited), whereas *B. longum* strains are resistant to oxygen availability (up to 83.5% were able to survive). Bacterial oxygen tolerance appears to be scientifically and industrially significant. Low tolerance will result in decreased activity or cell death already at the stage of preparing the bacteria for further stimulation in the experiment. This, in turn, may affect the altered eukaryotic cell response in the study model. The sensitivity of bacteria to oxygen may also affect the effectiveness of commercial probiotic formulation, for example, bacteria in the form of a live cell suspension may reduce their viability after opening. Therefore, determining how individual strains respond to oxygen availability seems to be one of the most important aspects of the conducted study. One of the control samples prepared in the research presented was bacteria suspended in MRS broth + L-cysteine but with oxygen presence. In comparison to the baseline (anaerobic condition, recommended for the *Bifidobacterium* culture) selected strain differed in oxygen resistance. Strain CCDM 219 (belonging to *B. longum)* indicated a decrease in the live population and an increase in mixed ones. On the contrary, strain CCDM 368 (belonging to *B. adolescentis*) indicated a high oxygen resistance and no oxygen impact on the viability of the cells was noted. In our research, oxygen did not contribute to the increase in the dead populations of none of the selected bacterial strains. We got divergent results compared to Andriantsoanirina et al. [31]. However, the research methodologies were different. In our experiments, the bacteria were exposed to aerobic conditions for 6 h, which did not affect their mortality, but in the case of strain CCDM 219 it impacted growth in a mixed population. A 48-h incubation could change these trends dramatically. On the other hand, Andriantsoanirina et al. based the bacterial viability assessment on the widely used CFU method, which allows for the indication of live cells only. Cells in the intermediate state (VBNC) might be characterised here as dead, which could give a false picture of their proper survival rate. The results obtained show that *Bifidobacterium* species differ in their properties and an individual approach to their oxygen-response characteristics is essential. In addition, data emphasises the need to standardise the research methodology to obtain more reproducible results. Therefore, in terms of the response to oxygen conditions and indirect estimation of the viability, we decided to evaluate the bacterial redox activity in various environments. However, as an expression of environmental adaptation, bacteria have developed various electron transport mechanisms [32], and bifidobacterial oxygen tolerance alters between species. Analysing the redox activity for strain CCDM 368, we obtained interesting results. The bacteria had some initial redox potential (baseline), which even increased in the presence of oxygen (MRS control sample). This find corresponds to the preserved high survival rate in the live / death experiment. The redox potential was also maintained for K18h (comparable to the baseline) but decreased for the remaining experiments. In fact, there was a noticeable tendency to reduce the redox potential in the 18 h, 3 h, and RPMI with TNF-α environments, which indicates that the CCDM 368 strain could be susceptible to an inflammatory environment. However, experiments with strain CCDM 219 led us to different conclusions. Our research indicated that the redox activity of strain CCDM 219 is higher in the baseline sample (MRS broth + L-cysteine, anaerobic condition) and stays comparable in the MRS control sample (MRS broth + L-cysteine but with oxygen presence). This phenomenon could be explained by the experimental condition and ability of this strain to produce a mucus-like layer. Bacteria were placed on the cell culture plate in a property medium but without stirring, therefore, by gravity, they could sink to the bottom of the well, where, in combination with a protective mucus-like layer, oxygen was not so accessible to them. Additionally, we noted that the redox activity decreased dramatically in all eukaryotic environments. Moreover, both RPMI milieus allowed to preserve some potential in comparison with the CaCo2/HT29 environments. These findings suggest that the eukaryotic component shapes the bacterial properties, and it could have an impact on its vitality. Indeed, the epithelial layer is the first line between the external and internal milieu. Epithelial cells are rich in pathogen recognition receptors (PRRs) which constantly sense microbial compounds and, moreover, are classified as non-professional phagocytes [33]. Epithelial cells may modify bacteria in a way in order to prepare them for cellular recognition or phagocytosis, thus affecting bacterial vitality. However, this venturesome statement demands further investigation.

We determined the bacterial membrane potential as well, and similarly to the above data, we noted a discrepancy between the selected strains. The membrane potential is a dynamic value and can fluctuate, including the cell's hyperpolarization and depolarization. The malleability of membrane potential allows bacteria for environmental adaptation and cell–cell signalling. It is crucial also in cell division, antibiotic resistance and homeostasis keeping [34, 35]. Our research indicated that strains CCDM 219 and CCDM 368 are characterised by a different membrane response to a changing environment. Strain CCDM 219 tended to raise the membrane potential under aerobic conditions (MRS control) compared to baseline. This is confirmed by our anterior observation. As signalled earlier, the hyperpolarization of bacterial membranes may be associated with a greater tendency to combine the PI and Syto 9 dyes in the viability assay and distinction of the mixed population. However, bacteria did not significantly change the membrane potential, which stayed comparable to the initial value, in experiments with eukaryotic cells and their culture medium. The additional mucus-like layer may protect bacteria against environmental factors. Different observation concerns strain CCDM 368. These bacteria do not change the membrane potential under aerobic conditions (MRS control) compared to baseline, however, they significantly decrease it in response to the cellular environment. Since the differences between the single groups were not observed, we conclude that culture medium RPMI impacts primarily bacterial behaviour. In fact, the cell culture medium used by us contained, among others, antibiotics (penicillin and streptomycin). *Bifidobacterium* are resistant to streptomycin, but sustainable for penicillin [36–38]. It has been also shown that cellular depolarization may be associated with the acquisition of greater resistance to antibiotics by some bacteria and their survival [39, 40]. Therefore, it seems that strain CCDM 368, through its membrane potential, reacts to the antibiotic presence in the environment and/or adapts to the environment to survive. It is worth emphasizing that experiments based on the stimulation of eukaryotic cells with bacteria are often performed in the presence of antibiotics, and this may affect the activity of bacteria and the results obtained.

The β-galactosidase, commonly known as lactase, is an important enzyme used in the pharmaceutical and food industries to alleviate symptoms of lactose intolerance and to produce specialised lactose-free foods [41, 42]. Common sources of obtaining this enzyme are plants, fungi and bacteria. Many strains of *Bifidobacterium*, including *B. adolenscentis* and *B. longum*, show the ability to produce it, which additionally increases their probiotic value [43, 44]. Therefore, we decided to check whether the selected strains produce this enzyme, and if so, whether its production is influenced by the external environment. The comparison of the two selected strains indicated that the bacteria differ in enzyme presence. At baseline, strain CCDM 219 indicated a high production of β-gal whereas strain CCDM 368 was characterized by a very low productivity. In the experimental milieu, strain CCDM 219 decreased significantly the number of positive cells in eukaryotic cells presence, but not in the RPMI controls. This indicates that the epithelial cells can shape the enzymatic activity of bacteria. Some analogy was observed also in strain CCDM 368, which significantly decreased the number of β-gal positive cells in the model of 'an active inflammatory state' (K18h and 18 h) and indicated the tendency to its reduction in remaining experiments, except the MRS control samples. Our observations (the effect of contact with eukaryotic cells on the reduction of enzyme production) may be an essential aspect of the effectiveness of lactase-based supplements. Since bacteria initially show an ability to enzyme production but decrease their efficiency in contact with eukaryotic cells, the supplement's effectiveness may closely depend on the number of bacteria used. On the other hand, too much bacterial supply and prolonged contact with the host cells can also be harmful, as shown in SRB test for 24 h. Therefore, this complex issue requires further research.

Our study has some limitations, for example, the inflammatory state was induced by TNF-α. It could happen, that using a different inflammatory stimulus, bacteria will respond in a different manner. Similarly, we used a complex RPMI 1640 medium only, the direction of response to the different cell culture medium and/or single component of culture media is unknown and/or demands further characterisation. Further studies are needed to determine the malleability of bacterial cells.

## Conclusion

In conclusion, our research showed that the experimental environment has an impact on *Bifidobacterium* cells. The knowledge of bacterial susceptibility to foreign environments may be crucial in the prediction of their probiotic attributes. In the in vitro experiments, bacteria have to come up against oxygen, cell culture medium, eukaryotic cells etc. In *in-vivo* experiments, bacteria are exposed to digestion acids, the presence of the host cells and other bacteria. Finally, in the commercial production of food supplements, bacteria must get through several technological processes. The external milieu can significantly impact the activity of the bacteria and the induced changes can abolish the beneficial effects. Therefore, strict standardisation of bacterial growth and profiling of bacteria malleability in response to different factors could be worthwhile. These preliminary steps can help to reduce the costs and appoint the strains, whose beneficial impact on the host is highly reproducible. Moreover, the literature data indicates that the field of bacterial vicissitudes remains unexplored. The probiotic bacteria-host studies mainly focus on the effects of the eukaryotic component. Bacteria are perceived rather as inert players, while the available transcriptomic analysis clearly indicates bacterial reactivity and dependence on the milieu [45, 46]. The transcriptomic analysis of bacterial response in conjunction with phenotypic changes determination could help to better understand the basic processes underlying between bacteria community as well as bacteria-host interaction.

## Methods

### Cells culture

#### Bacteria culture

*Bifidobacterium* strains were obtained from the Czech Collection of Dairy Microorganisms (CCDM, Laktoflora, Milcom, Tábor, Czech Republic). The *Bifidobacterium adolescentis* CCDM 368 (CCDM 368, origin: human feces*) and Bifidobacterium longum* ssp. *longum* CCDM 219 (CCDM 219; origin: infant feces) were grown in De Man, Rogosa and Sharpe broth** (**MRS broth) (Oxoid) liquid medium with 0.05% L-cysteine (Serva) at 37 °C under anaerobic conditions using Genbox Anaer (BioMerieux). Before experiments, bacteria were counted with the use of a spectrophotometric microplate counter (BioTek, Winooski, Vermont, USA) at OD_600_ (which corresponds to the number of CFUs on MRS agar plates after 48 h of growth under anaerobic conditions) and washed with phosphate-buffered saline (PBS, pH 7.4) (10 min, 6,000 × g). A suspension of 10^8^ colony-forming unit (CFU) bacteria in 50 µl PBS was used in further experiments.

#### Eukaryotic cells culture

##### CaCo-2 and HT-29 cell lines culture

The cell line CaCo-2 (HTB-37) was obtained from ATCC (American Type Culture Collection). The cell line CaCo-2 was cultured on Dulbecco′s Modified Eagle′s Medium—high glucose (DMEM) medium (Sigma-Aldrich) supplemented with 10% (v/v) fetal bovine serum (FBS) (Gibco), 1% (v/v) mixture of penicillin (100 U/ml) and streptomycin (100 µg/ml) (Sigma-Aldrich), 1% (v/v) non-essential amino acids (Gibco) and 1 mM N-2-hydroxyethyl piperazine-N-2-ethane sulfonic acid (HEPES) (Gibco). The cell line HT-29 (HTB-38) was obtained from ATCC. The cell line HT-29 was cultured on Dulbecco's Modified Eagle Medium/Nutrient Mixture F-12 (DMEM/F12) medium (Gibco) supplemented with FBS and penicillin/ streptomycin as previously. Both cultures were incubated at 37 °C, with 5% CO_2_. One week before experiments, both cell lines were incrementally accustomed to Roswell Park Memorial Institute 1640 Medium (RPMI 1640) medium (Gibco) supplemented with FBS and penicillin/streptomycin as previously.

##### CaCo-2 and HT-29 co-culture

The co-culture was established following Ferraretto et al. [47] with minor changes. Briefly, both cell lines on the RPMI 1640 medium were removed from their culture bottle by trypsinization, washed (5 min, 200 × g) and counted with the use of an automatic cell counter (TC, BioRad). The mixture of Caco-2 and HT-29 (CaCo2/HT29) in a ratio 2:1 (0.2 × 10^6^ CaCo-2 and 0.1 × 10^6^ HT-29 cells) was seeded on 24 wells plate (ThermoScientific) in 1 ml of complete RPMI 1640 medium. The cultivation was continued until the demanded confluence was reached on the wells. Then, stimulation was started according to the planned models.

### Models of experiments

Bacterial malleability was determined in response to the environment created by eukaryotic cells in two different models:

1. Active inflammatory state

CaCo2/HT29 cells mixture was seeded on the 24 wells plate (0.3 × 10^6^ cells/ml in complete RPMI 1640 medium) and cultivated within to 85% confluence achievement (average 2–3 days), with daily medium replacement. Then, the inflammatory state was induced by adding 10 ng/ml tumor necrosis factor α (TNF-α) (Abcam) and cells were further incubated for an additional 18 h. Finally, the amount of 10^8^ CFU/ml of *Bifidobacterium adolescentis* CCDM 368 and *Bifidobacterium longum* ssp. *longum* CCDM 219 was added to inflammatory state cells (18 h) and appropriate controls: co-culture CaCo2/HT29 without inflammatory state (K18h), complete cell culture medium (RPMI), complete cell culture medium with the addition of the inflammatory factor (RPMI + TNF-α) and MRS broth under aerobic conditions (MRS).

2. Protection against inflammatory state

CaCo2/HT29 cells mixture was seeded on the 24 wells plate as previously and cultivated to full confluence achievement (average 3 days). Then, the amount of 10^8^ CFU/ml of *Bifidobacterium adolescenti*s CCDM 368 and *Bifidobacterium longum* ssp. *longum* CCDM 219 was added to co-culture and appropriate controls (co-culture CaCo2/HT29 without inflammatory state (K3h), complete cell culture medium (RPMI), complete cell culture medium with the addition of the inflammatory factor (RPMI + TNF-α) and MRS broth under aerobic conditions (MRS). After 3 h of stimulation, the inflammatory state inductor (10 ng/ml TNF-α) was added to the proper wells.

The description of experimental groups and samples are shown in Table 1. The scheme of experiments is presented in Additional file 5.

**Table 1:** The experimental groups and samples

**Model 1: Active Inflammatory state ( inflammatory state induced 18 h before the bacterial stimulation)**	
* K18h*	bacteria in co-culture with CaCo2/HT29 (control)
* 18 h*	bacteria in co-culture with CaCo2/HT29 in inflammatory state induced 18 h prior bacteria addition
**Model 2: Protection against the inflammatory state (inflammatory state induced 3 h after bacterial stimulation)**	
* K3h*:	bacteria in co-culture with CaCo2/HT29 (control)
* 3 h*:	bacteria in co-culture with CaCo2/HT29 in inflammatory state induced 3 h after bacteria addition
**Baseline and shared controls**	
* Baseline*	bacteria in MRS medium under anaerobic conditions (conditions recommended for *Bifidobacterium* culture)
* RPMI*	bacteria in RPMI 1640 medium
* RPMI* + *TNF-α*	bacteria in RPMI 1640 medium with the addition of an inflammatory factor
* MRS medium*	bacteria in MRS medium under aerobic conditions

### Bacterial-based experiments

#### TEM (*Transmission electron microscopy)* and EDS (energy dispersive spectroscopy) measurements

Bacteria were added to proper wells according to experimental model assumptions and incubated for 6 h (5% CO_2_, 37 C). After stimulation, bacteria together with CaCo2/HT29 cells were removed from the wells by trypsinisation and washed in PBS (5 min, 200 × g). Bacteria suspended in cell culture media/broth were washed with PBS only (without trypsinization). Next, the cells were fixed in 2.5% pH 7.2 buffered glutaraldehyde and stored at 4 C for further preparation. Then cells were contrasted with 2% osmium tetroxide in the dark. The material was washed with a buffer and contrasted with 2% uranyl acetate for 12 h. Then the samples were passed through an ascending alcohol series of 30%, 50%, 70%, 90%, 96% and 99.8%. The material prepared in this way was embedded in a medium-hard epoxy resin. After polymerization, ultra-thin sections were prepared on an ultramicrotome (Leica). Sections of 60 nm were prepared from the resin blocks and placed on copper grids (400 Mesh) with formvar film and carbon coating. Imaging was performed using a JEOL JEM-F200, JEOL Japan microscope. Elemental EDS analysis was performed using a JEOL microscope. Spectra analysis was carried out in the analysis program JED Series. In experiments, the model with bacteria in an active inflammatory state was used exclusively. The pictures obtained were estimated by two independent researchers (subjective observation).

#### Bacterial live-dead analysis

The commercially available kit Live/Dead BacLight Bacterial Viability and Count Kit (Molecular Probes) was used according to manufacturer procedure. Briefly, after 6 h of stimulation, the cell medium was collected from the wells and the CaCo2/HT29 layer was gently rinsed with warm 0,85% sodium chloride (NaCl). Then both fractions were combined and centrifuged (8 min, 3,500 × g). Finally, the bacterial pellet was washed in NaCl (8 min, 3,500 × g), suspended in 1 ml of NaCl with propidium iodide (PI) and Syto9 dyes in a ratio of 1:1 and incubated for 10 min at room temperature (RT) in the dark. Next, the samples were read with the use of a CytoFlex cytometer (Backman Coulter). The flow cytometry data collection (5000 events) was based on a blue laser with 488/8, 525/40, and 610/20 nm filters. The bacterial population was gated based on forward and side scatters (FSC-H/SSC-H) and logarithmic axis.

#### Redox activity analysis

The redox activity tests were prepared based on the commercial kit (BacLight RedoxSensor CTC Vitality Kit, Molecular Probes) according to the manufacturer's guidelines. Briefly, after 6 h of stimulation, the cell medium was collected from the wells and the CaCo2/HT29 layer was gently rinsed with warm PBS. Then both fractions were combined and centrifuged (8 min, 3500 × g). Finally, the bacterial pellet was washed in PBS (8 min, 3,500 × g) and bacteria were suspended in 50 mM 5-cyano-2,3-ditolyl tetrazolium chloride (CTC) solution and incubated for 30 min protected from light. Next, the samples were washed twice in PBS (8 min, 3,500 × g) and fixed in 2% paraformaldehyde (PFA) (10 min). After fixation, the samples were washed, suspended in 1 ml of PBS and kept in the fridge before the flow cytometry analysis. The flow cytometry data collection (10,000 events) was based on a blue laser with 488/8 and 610/20 nm filters. The bacterial population was gated as previously.

#### Bacterial membrane potential

The membrane potential tests were prepared based on the commercial kit (BacLight Bacterial Membrane potential, Molecular Probes) according to the manufacturer's procedures with minor changes. Briefly, after 6 h of stimulation, the cell medium was collected from the wells and the CaCo2/HT29 layer was gently rinsed with warm PBS. Then both fractions were combined and centrifuged (8 min, 3,500 × g). Finally, the bacterial pellet was washed in PBS (8 min, 3,500 × g) and bacteria were suspended in 1 mM EDTA and 3 mM 3,3’-diethyloxa-carbocyanine iodide (DiOC_2_(3)) solution in PBS on the sterile 96 wells dark plate. After 10 min of incubation at 37 °C, the samples were read with the use of the ClarioStar fluorescence reader at 37 °C with filters for FITC and Texas Red (measurement in the time point).

#### Bacterial β-galactosidase (β-gal)

The β-gal tests were prepared based on the commercial kit (CellEvent Senescence Green Flow Cytometry Assay Kit, Invitrogen) adapted for the bacteria-based experiments. Briefly, after 6 h of stimulation, the cell medium was collected from the wells and the CaCo2/HT29 layer was gently rinsed with warm PBS. Then both fractions were combined and centrifuged (8 min, 3,500 × g). Finally, the bacterial pellet was washed in PBS (8 min, 3,500 × g) and bacteria were suspended in 2% PFA and incubated for 10 min protected from light. Next, the samples were washed twice in PBS (8 min, 3,500 × g) and stained with CellEvent Senescence Green Probe reagent diluted 1:500 in working buffer (1 h, 37 °C, protected from light and without CO_2_ additional sources). Then, the samples were washed, suspended in 1 ml of PBS and kept in the fridge before the flow cytometry analysis. The flow cytometry data collection (10,000 events) was based on a blue laser with 488/8 and 610/20 nm filters. The bacterial population was gated as previously.

### CaCo2/HT29 based experiments

#### Cell proliferation

Cell proliferation was determined based on Sulforhodamine B (SRB) assay. Briefly, 3 × 10^4^ of CaCo2/HT29 co-culture (ratio 2:1) in 100 µl RPMI complete medium was seeded on 96-well plate 24 h prior to bacterial stimulation. Then, the amount of 10^6^, 10^7^, and 10^8^ CFU of CCDM 219 and CCDM 368 in 100 µl RPMI 1640 complete medium was added to the eukaryotic cells. After 6 h and 24 h of costimulation, the 10% cold trichloroacetic acid (TCA) was added to each well and incubated for 1 h at 4 °C. Next, the supernatant was removed, and the plate was washed 3 times with distilled water and dried in the air. Then, 0.06% SRB was added to the wells and incubated for 30 min in darkness, at RT. The dye excess was removed from the wells by washing in 1% acetic acid. To dissolve the remaining SRB, 10 mM Tris–HCl (pH 10.5) was used. The absorbance was read at 510 nm.

### Data analysis

The FlowJo VX.07 software (Tree Star Inc, USA) was used for the analysis of flow cytometric data. Experiments were done in at least two biological and three technical repetitions. Statistical analyses were performed with GraphPad software (Prism). Normality was determined using the Shapiro–Wilk test. The analysis of the differences between the baseline and the other groups was performed using the One-way ANOVA test. Differences between groups in individual models were assessed based on a paired t-test. Correlations were analyzed based on Pearson's correlation coefficient of linear regression (r). The level of significance was *p* < 0.005.

### Supplementary Information

**Additional file 1:** **Fig. S1.** Dots plots of CCDM 368 and CCDM 219 viability.

** Additional file 2:** **Fig. S2.** The redox activity of CCDM 219 and CCDM 368 dot plots.

** Additional file 3:** **Fig. S3.** Bacterial response to the  environment with and without antibiotics.

** Additional file 4:** **Fig. S4.** β-gal production by CCDM 219 and CCDM 368 dot plots.

** Additional file 5:** **Fig. S5.** Experimental models.

## Data Availability

All relevant data and materials that support the findings of this study are available from the corresponding author upon request.

## Abbreviations

ATCC: American Type Culture Collection
CCDM: Culture Collection of Dairy Microorganisms
CFU: Colony forming unit
CTC: 5-Cyano-2,3-ditolyl tetrazolium chloride
Cxcl2: C-X-C Motif Chemokine Ligand 2
DiOC2(3): 3,3’-Diethyloxa-carbocyanine iodide
DMEM/F12: Dulbecco's Modified Eagle Medium/Nutrient Mixture F-12
DMEM: Dulbecco′s Modified Eagle′s Medium—high glucose
EDS: Energy dispersive spectroscopy
EPS: Exopolysaccharides
FBS: Fetal bovine serum
Fpr: Formylated peptide receptors
HEPES: N-2-hydroxyethyl piperazine-N-2-ethane sulfonic acid
IBD: Inflammatory bowel disease
MRS: De Man, Rogosa and Sharpe broth
NaCl: Sodium chloride
NLRP3: NOD-, LRR- and pyrin domain-containing protein 3
PBS: Phosphate-buffered saline
PFA: Paraformaldehyde
PI: Propidium iodide
PRRs: Pathogen recognition receptors
RPMI 1640: Roswell Park Memorial Institute 1640 Medium
RT: Room temperature
SRB: Sulforhodamine B
TCA: Trichloroacetic acid
TEM: Transmission electron microscopy
TLR: Toll-like receptors
TNF-α: Tumor necrosis factor α
VBNC: Viable but non-culturable
β-gal: β-Galactosidase
