# Supplementary material for: What happens to Bifidobacterium adolescentis and Bifidobacterium longum ssp. longum in an experimental environment with eukaryotic cells?
Source: BMC Microbiol. 2024 Feb 19;24:60. doi: 10.1186/s12866-023-03179-z (PMC10875879; doi:10.1186/s12866-023-03179-z)
Supplement: Supplementary file 1 — Additional file 1: Fig. S1. Dots plots of CCDM 368 and CCDM 219 viability. [file 12866_2023_3179_MOESM1_ESM.docx]

Fig. S1. Dots plots of CCDM 368 and CCDM 219 viability.


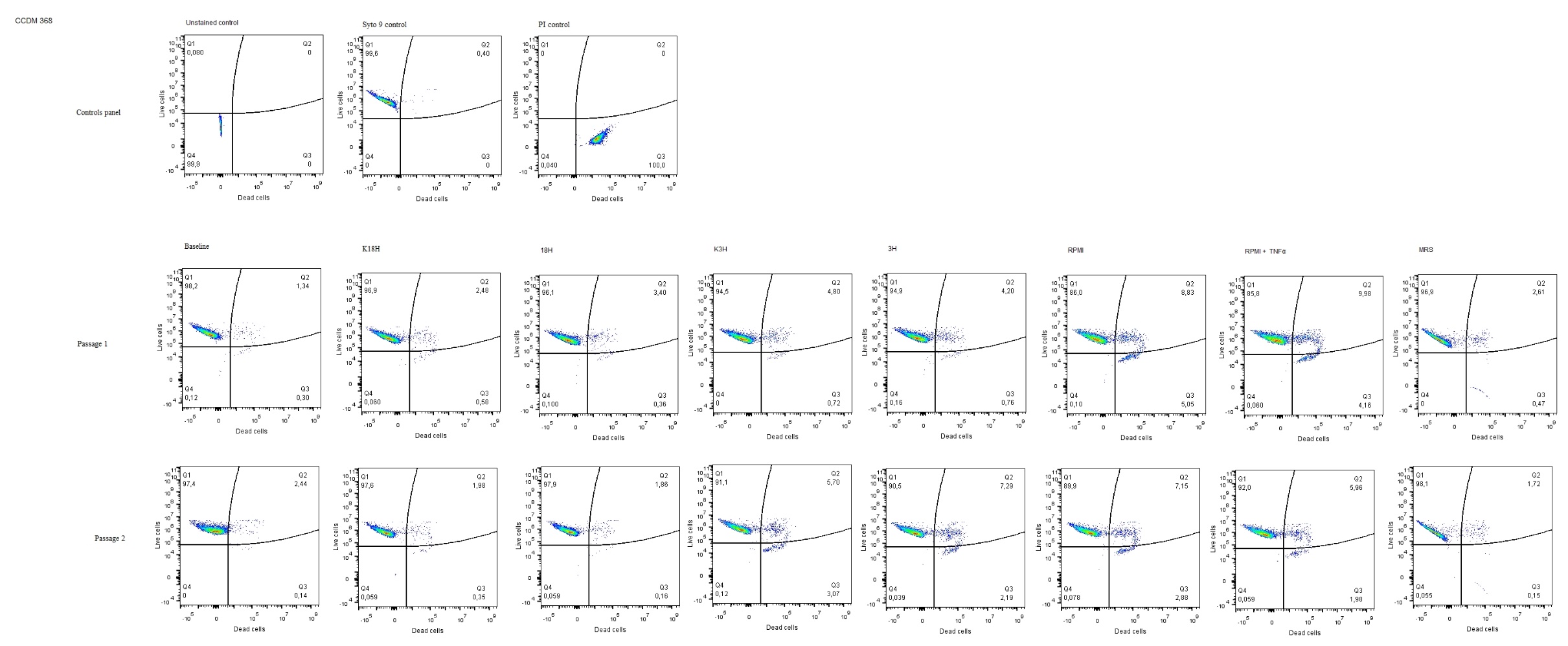
a) Examples of dot-plots for strain CCDM 368. Two different bacterial passages.


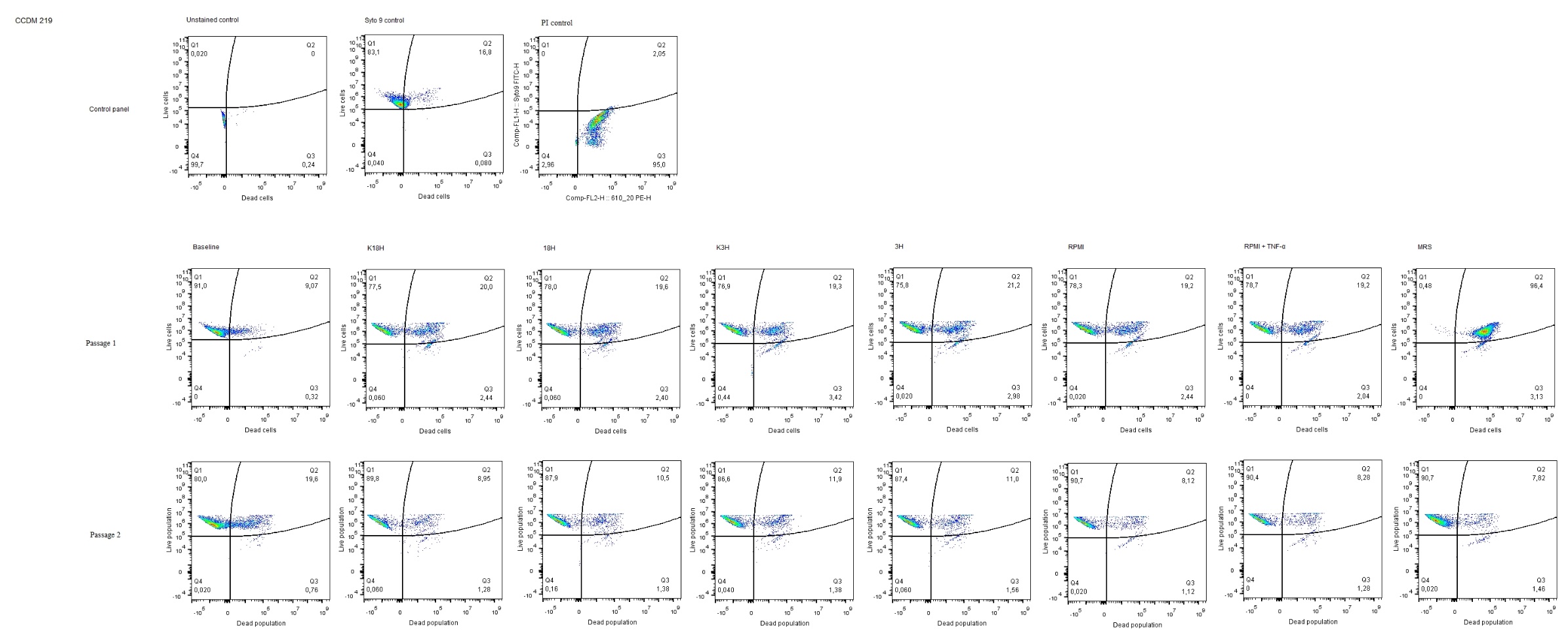
b) Examples of dot-plots for strain CCDM 219. Two different bacterial passages.
