# Supplementary figures and images for: What happens to Bifidobacterium adolescentis and Bifidobacterium longum ssp. longum in an experimental environment with eukaryotic cells?
Source: BMC Microbiol. 2024 Feb 19;24:60. doi: 10.1186/s12866-023-03179-z (PMC10875879; doi:10.1186/s12866-023-03179-z)

Fig. S2.
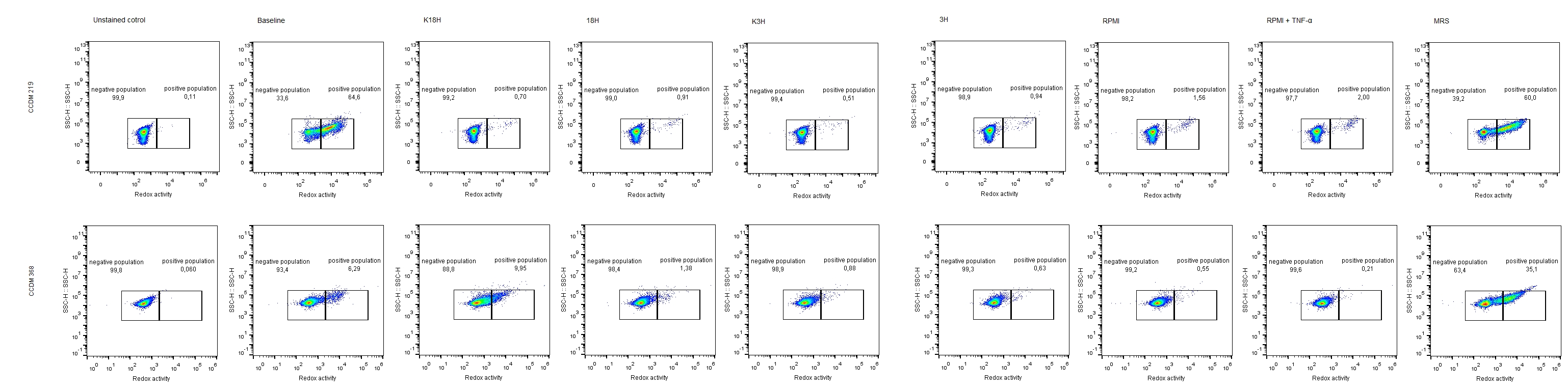
 The redox activity of CCDM 219 and CCDM 368 dot plots.

Supplement: Supplementary file 2 — Additional file 2: Fig. S2. The redox activity of CCDM 219 and CCDM 368 dot plots. [file 12866_2023_3179_MOESM2_ESM.docx]

Fig. S4. β-gal production by CCDM 219 and CCDM 368 dot plots.


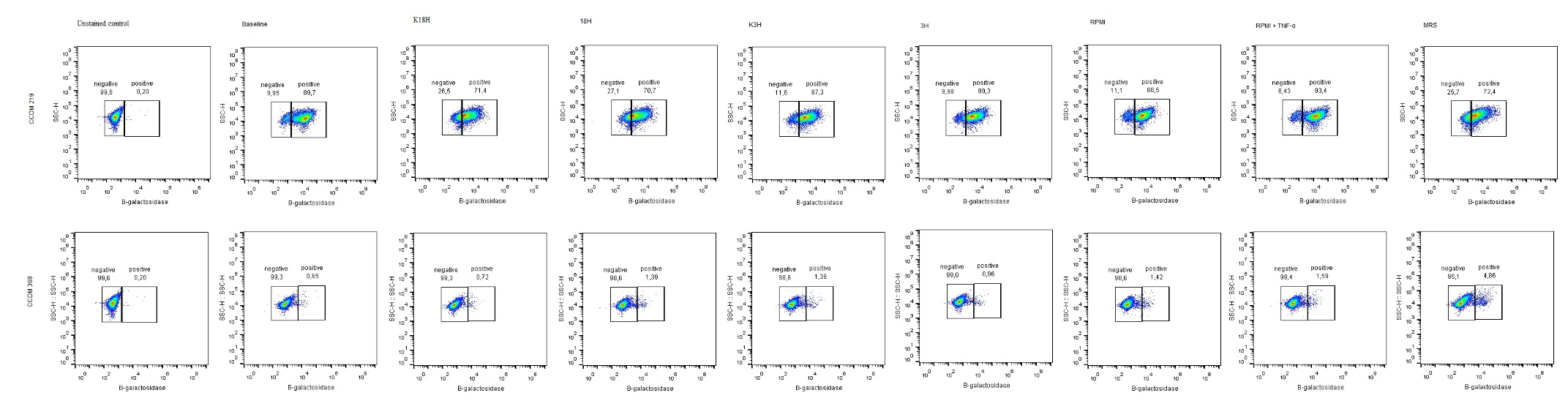

Supplement: Supplementary file 4 — Additional file 4: Fig. S4. β-gal production by CCDM 219 and CCDM 368 dot plots. [file 12866_2023_3179_MOESM4_ESM.docx]
