# Supplementary material for: What happens to Bifidobacterium adolescentis and Bifidobacterium longum ssp. longum in an experimental environment with eukaryotic cells?
Source: BMC Microbiol. 2024 Feb 19;24:60. doi: 10.1186/s12866-023-03179-z (PMC10875879; doi:10.1186/s12866-023-03179-z)
Supplement: Supplementary file 3 — Additional file 3: Fig. S3. Bacterial response to the environment with and without antibiotics. [file 12866_2023_3179_MOESM3_ESM.docx]

Fig. S3. Bacterial response to the environment with and without antibiotics

1. Membrane potential

1. Viability
