# Supplementary material for: What happens to Bifidobacterium adolescentis and Bifidobacterium longum ssp. longum in an experimental environment with eukaryotic cells?
Source: BMC Microbiol. 2024 Feb 19;24:60. doi: 10.1186/s12866-023-03179-z (PMC10875879; doi:10.1186/s12866-023-03179-z)
Supplement: Supplementary file 5 — Additional file 5: Fig. S5. Experimental models. [file 12866_2023_3179_MOESM5_ESM.docx]

Fig.S.5. Experimental models.


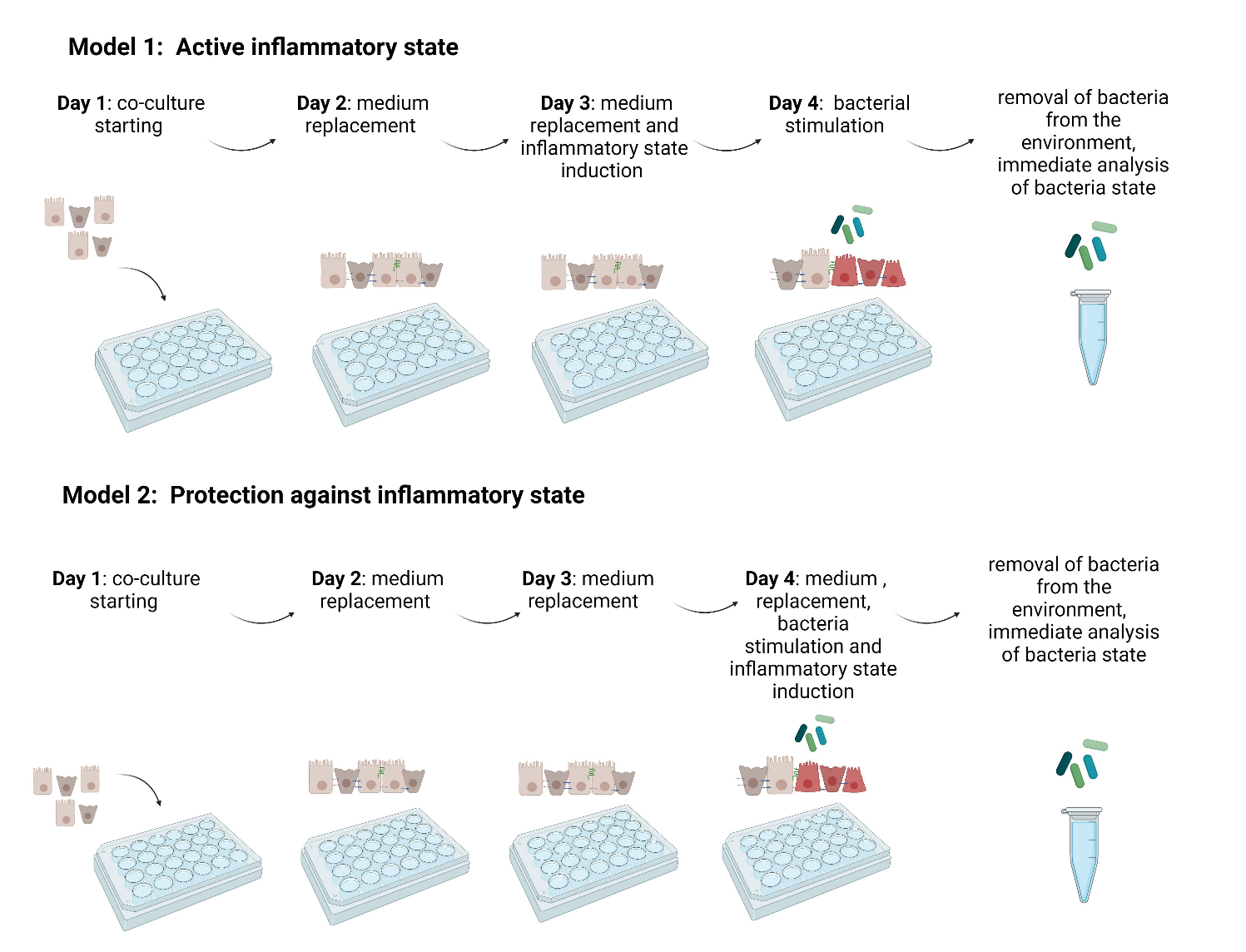


Model 1: Active inflammatory state (inflammatory state induced 18 h before the bacterial stimulation). The tested samples were as follows: *Baseline*: bacteria in MRS medium under anaerobic conditions, *K18h*: bacteria in co-culture with CaCo2/HT29 (control), *18h*: bacteria in co-culture with CaCo2/HT29 in inflammatory state induced 18 h prior bacteria addition, *RPMI:* bacteria in RPMI 1640 medium (control), *RPMI + TNF-α*: bacteria in RPMI 1640 medium with the addition of an inflammatory factor (control) and MRS: bacteria in MRS medium under aerobic conditions (control).

Model 2: Protection against the inflammatory state (inflammatory state induced 3h after bacterial stimulation). The tested samples: *Baseline*: bacteria in MRS medium under anaerobic conditions (conditions recommended for *Bifidobacterium* culture), *K3h*: bacteria in co-culture with CaCo2/HT29 (control), *3h*: bacteria in co-culture with CaCo2/HT29 in inflammatory state induced 3 h after bacteria addition, *RPMI:* bacteria in RPMI 1640 medium (control), *RPMI + TNF-α*: bacteria in RPMI 1640 medium with the addition of an inflammatory factor (control) and MRS: bacteria in MRS medium under aerobic conditions (control).
